# Integrative genomic and proteomic analyses identify candidate genetic loci and genetically supported proteins in age-related macular degeneration

**DOI:** 10.1186/s40662-026-00502-3

**Published:** 2026-08-14

**Authors:** Ning Chen, Yukang Liu, Zejun Chen, Yishen Xiang, Honghua Yu

**Affiliations:** 1 The Second School of Clinical Medicine, Zhujiang Hospital, Southern Medical University, Guangzhou, Guangdong China; 2 Department of Electrical and Electronic Engineering, The University of Hong Kong, Hong Kong, China; 3 Guangdong Eye Institute, Department of Ophthalmology, Guangdong Provincial People’s Hospital (Guangdong Academy of Medical Sciences), Southern Medical University, Guangzhou, China; 4 Joint Shantou International Eye Center, Shantou University and the Chinese University of Hong Kong, Shantou, 515041 Guangdong China

**Keywords:** Age-related macular degeneration, Genome-wide association study, Mendelian randomization, Proteomics, Druggable targets, Multi-omics integration, Immune mediation, Single-cell RNA sequencing

## Abstract

**Background:**

Age-related macular degeneration (AMD) is a leading cause of visual impairment; however, robust drug targets for primary prevention remain elusive. This study aimed to identify candidate genetic loci and putative genetically supported proteins for AMD through integrative genomics and proteomics.

**Methods:**

A genome-wide association study (GWAS) meta-analysis (28,543 cases and 1,072,092 controls) was performed to identify risk loci, followed by proteome-wide Mendelian randomization (MR), colocalization, and multi-omics summary-data-based MR with heterogeneity in dependent instruments (SMR-HEIDI) analyses to infer genetically supported proteins. Functional characterization included gene enrichment, single-cell RNA sequencing, mediation across 731 immunophenotypes, and a mouse knockout assessment. Druggability was evaluated using the Drug–Gene Interaction Database.

**Results:**

The GWAS meta-analysis identified 10 candidate genetic loci for AMD (including three previously reported and seven newly identified loci). Proteome-wide MR and colocalization analyses uncovered 23 genetically supported plasma proteins (11 risk and 12 protective factors). Multi-omics SMR-HEIDI analysis prioritized *LAMC2* and *PLTP* as first-tier risk genes, which was consistently supported across protein, expression, and methylation levels. Single-cell RNA sequencing revealed cell-type-specific enrichment of key genes in monocytes and stromal/progenitor-like cells within AMD retinas, and subsequent mediation analysis identified specific immune traits, most notably regulatory T-cell subsets, as potential partial mediators of AMD risk. Druggability assessment identified 15 target genes, and bioinformatic enrichment nominated cyclophosphamide. Given the toxicity profile of cyclophosphamide, this computational finding serves strictly as a pathway indicator, reinforcing the broader therapeutic relevance of modulating the identified immune-related targets.

**Conclusion:**

Our integrative analyses identified candidate genetic loci and genetically supported proteins for AMD, providing a foundation for translating genomic findings toward primary prevention. The exploratory nature of this study highlights the need to experimentally validate the proposed targets and underlying mechanisms.

**Supplementary Information:**

The online version contains supplementary material available at 10.1186/s40662-026-00502-3.

## Background

Age-related macular degeneration (AMD) is a leading cause of global visual impairment, primarily affecting adults over 55 years of age. An estimated 196 million people worldwide have AMD, which substantially reduces their quality of life and can lead to blindness. AMD is characterized by progressive central vision loss and can severely impair daily functioning, thus posing a major global public-health burden [1]. The disease comprises two subtypes: neovascular and atrophic AMD. Although treatments for the neovascular form have advanced, effective therapies for atrophic AMD remain limited, making this subtype a pressing global public-health issue [2, 3].

Previous studies have indicated that genetic factors contribute to 40%–60% of AMD risk [4]; therefore, human genetics can help mitigate residual confounding and reverse causation [5], and large-scale genomic analyses can provide valuable insights into the causal mechanisms of complex traits such as AMD [6]. International consortia, such as the AMD Genomics Consortium, UK Biobank, and FinnGen, have identified multiple AMD-associated genetic loci, implicating pathways involving complement activation, lipid metabolism, angiogenesis, immunity, and cellular motility [7, 8]. Proteins involved in metabolic and complement pathways are thought to influence AMD onset and progression, with some complement components already serving as targets for recently approved therapies [9]. Integrating proteomic data into Mendelian randomization (MR) studies can help clarify the genetic basis of diseases influenced by circulating factors [10], and causal evidence supporting circulating proteins as promising drug targets [11] could open avenues for exploring whether genetic findings might eventually inform preventive strategies for AMD. However, the mechanisms through which these proteins modulate immune responses and drive disease progression remain unclear. In particular, the immune cascade initiated by circulating proteins in AMD, which potentially involves immune-cell recruitment and regulation, represents a key unresolved question [12]. Deeper mechanistic studies are essential to clarify the protein–AMD relationship and generate hypotheses for candidate interventions.

In this study, we conducted a large-scale genome-wide association study (GWAS) meta-analysis incorporating four datasets to enhance statistical power for locus discovery. We then integrated these GWAS summary statistics with protein quantitative trait loci (pQTLs) obtained from the deCODE Genetics and UK Biobank Pharma Proteomics Project (UKB-PPP) studies to conduct MR and genetic colocalization analyses. To evaluate the biological credibility of our genetic findings in an exploratory manner, we performed extensive downstream analyses, including multi-omics integration, single-cell RNA sequencing (scRNA-seq), and mediation analysis. This “staged, cross-population, multi-phenotype” strategy facilitates the discovery of candidate AMD risk genes and generates testable hypotheses for future mechanistic and therapeutic studies on AMD.

## Methods

### Data sources

#### GWAS data sources

A summary of the included GWAS datasets, with details on sample sizes, phenotype definitions, and study populations, is provided in Additional file 1: Table S1. The study population for the meta-analysis included 1,100,635 participants, which were derived from the FinnGen Release 10 AMD GWAS, the AMD GWAS by Jiang et al. [13].

Included Dataset 1: Early AMD GWAS [14]. The meta-analysis for early AMD (comprising 11 studies, totaling 14,034 cases and 91,214 controls) utilized an inverse-variance weighted (IVW) fixed-effects model and incorporated data from the International Age-Related Macular Degeneration Genomics Consortium (IAMDGC) and UK Biobank. Included Dataset 2: AMD data from the FinnGen Study [15]. Initiated in 2017, the FinnGen project (http://www.finngen.fi/en) encompassed 9271 AMD cases and 381,339 controls, providing GWAS summary statistics for AMD. Included Datasets 3 and 4: GWAS cohorts from Jiang et al. [13]. Jiang et al. employed a fastGWA-GLMM model-based tool for the genome-wide association analysis of genetic data derived from 456,348 individuals of European ancestry in the UK Biobank. The GWAS summary statistics for AMD included self-reported macular degeneration (3493 AMD cases and 144,486 controls) and retinal macular degeneration (1295 AMD cases and 455,053 controls).

Although the included GWAS datasets captured different stages and definitions of AMD, such as early AMD, clinically diagnosed AMD, and self-reported macular degeneration, they collectively represent the broader disease spectrum of AMD. Extensive evidence supports a shared genetic architecture across these phenotypes, with major AMD risk loci (e.g., *CFH*, *ARMS2*/*HTRA1*, *C3*, and *CFI*) consistently implicated across early and advanced disease stages, as well as across clinically confirmed and self-reported cases [6, 7]. Previous large-scale AMD GWAS meta-analyses have similarly integrated heterogeneous case definitions to enhance statistical power for locus discovery [6, 8]. To empirically validate this shared genetic architecture within our specific datasets and address potential phenotypic heterogeneity, we performed a genome-wide genetic correlation analysis using linkage disequilibrium score regression (LDSC) and cross-cohort effect direction consistency checks for lead variants. Therefore, combining these datasets under a unified AMD framework is methodologically justified and aligns with established practices in the field.

Datasets 1, 3, and 4 include participants from the UK Biobank, leading to a potential sample overlap across the GWAS datasets. However, Dataset 1 is derived from a meta-analysis of 11 studies (including the IAMDGC and UK Biobank), whereas Datasets 3 and 4 are from distinct sub-phenotypes (self-reported and retinal macular degeneration) analyzed separately. To capture a broader disease spectrum, our meta-analysis integrated datasets encompassing distinct AMD sub-phenotypes (early AMD, self-reported macular degeneration, and advanced retinal degeneration). While this approach introduces a theoretical degree of sample overlap among the UK Biobank participants, the distinct phenotypic definitions inherently minimize the intersection of actual AMD cases. Notably, the robustness of our approach is objectively supported by our genomic inflation factor (λ = 1.052), which demonstrates that this partial cohort overlap preserved strict type I error control without the artificial inflation of test statistics [16, 17]. To rigorously address the potential bias from sample overlap, we further evaluated the LDSC intercept, which remained close to unity (intercept = 0.9972, standard error [SE] = 0.0232), indicating that the test statistic inflation primarily reflected polygenic architecture rather than confounding from sample overlap or population stratification[18]. Furthermore, the high consistency of findings within the independent FinnGen cohort served as an additional sensitivity analysis to mitigate the influence of UK Biobank-specific overlap. The distinct phenotypic ascertainment across these datasets inherently minimizes the effective overlap in case populations.

#### Plasma proteomics data sources

The deCODE Genetics study generated genome-wide data through the direct genotyping of 49,708 Icelandic individuals using Illumina single nucleotide polymorphism (SNP) chips, which served as a reference panel for subsequent genotype imputation. This imputation process was applied to an expanded cohort of 166,281 individuals, resulting in over 27 million imputed variants for downstream analyses [19]. Plasma protein levels were measured in 35,559 participants. The pQTLs were identified via genome-wide association testing adjusted for 4907 aptamer levels. The UKB-PPP database (https://www.synse.org/Synapse:syn51364943) characterized plasma protein data for 54,219 participants and mapped pQTLs for 2940 proteins. The pQTLs located near their homologous gene loci are termed *cis*-pQTLs. For the proteome-wide MR analysis, we selected *cis-*acting SNPs (within ± 1 Mb of the target gene region) as instrumental variables (IVs) separately within the deCODE and UKB-PPP datasets. For each dataset, we retained SNPs that were significant (*P* < 5 × 10^−8^) and independent (linkage disequilibrium [LD] R^2^ < 0.1). The two datasets were analyzed independently, with deCODE serving as the primary discovery dataset and UKB-PPP used for replication; thus, a protein was considered to have supported evidence if it showed significant association in the deCODE analysis and a consistent direction of effect in the UKB-PPP analysis, rather than requiring both datasets to contribute IVs for the same protein simultaneously.

#### Sources for transcriptomic and DNA methylation QTL data

Gene expression data were obtained from the eQTLGen Consortium [20], which provided *cis-*acting expression QTL (*cis*-eQTL) summary statistics derived from 31,684 samples for genes corresponding to plasma protein targets. Tissue-specific *cis*-eQTL data obtained from 49 GTEx (v8) tissue samples (n = 15,201) were also included to assess tissue-specific expression patterns and potential off-target effects [21]. All expression datasets were based on publicly available summary statistics. Blood methylation QTL (mQTL) data were obtained from McRae et al. [22], which included 1980 Europeans and employed the Illumina HumanMethylation450K BeadChip microarray. After background correction, the associations between SNPs and CpG sites were tested using a generalized linear model with a logistic link function adjusted for probe-specific effects.

#### Immune cell data sources

This study leveraged a compiled collection of 731 immunological traits from an extensive GWAS Catalog, spanning the accession numbers from GCST0001391 to GCST0002121 [23]. The 731 immune traits encompass a range of phenotypes, with approximately 26% representing relative cell counts, 16% consisting of absolute cell counts, and 4% dedicated to morphological parameters.

### Genetic locus discovery and central GWAS analysis

Following the Meta-analysis of Observational Studies in Epidemiology reporting guidelines, we performed a comprehensive meta-analysis using METAL software (https://genome.sph.umich.edu/wiki/METAL), applying both fixed- and random-effects models to account for potential heterogeneity. Only SNPs with a minor allele frequency (MAF) > 1% were retained to focus on common variants, enhance statistical power, and reduce false positives. The genomic inflation factor was calculated to assess potential bias, and Manhattan plots of genome-wide significant loci generated using the rMVP R package (https://github.com/xiaolei-lab/rMVP).

Gene prioritization, using both the nearest gene to the index variant and the polygenic priority score (PoPS) algorithm, was specifically conducted for the 10 prioritized candidate loci (located > 500 kb from known signals) to nominate their most likely causal genes [24]. PoPS integrates GWAS summary statistics with gene expression, biological pathways, and protein–protein interaction data to improve genetically supported gene nomination at noncoding loci. For each locus, we annotated genes within a 500-kb window and selected the gene with the highest PoPS score as the prioritized candidate. Candidate loci were defined as those located more than 500 kb from any previously reported index variant, which is a widely adopted approach used in recent large-scale GWAS meta-analyses [25].

To rigorously determine whether the candidate loci were independent of previously reported AMD signals, we conducted a conditional and joint analysis (COJO) using GCTA (version 1.93.2). We conditioned our meta-analysis summary statistics on known AMD index SNPs, using the 1000 Genomes Project Phase 3 European subpopulation as the LD reference panel. Loci that maintained genome-wide significance (conditional *P* < 5 × 10^−8^) after conditioning were defined as independent, newly identified loci.

### Functional validation of candidate AMD genetic loci

We leveraged an MR phenome-wide association study (MR-PheWAS) as a supplementary exploratory approach to systematically evaluate the pleiotropic profiles of candidate AMD loci. This assessment of systemic associations complemented our primary findings by providing foundational insights into shared biological networks and potential off-target effects. Diverse trait GWAS summary statistics for the MR-PheWAS were extracted from the IEU OpenGWAS project, which comprises over 40,000 GWAS summary datasets. To explore associations between the candidate AMD loci and these diverse traits, we utilized the ieugwasr (version 0.2.2) R package (https://github.com/MRCIEU/ieugwasr). A significance threshold of *P* < 5 × 10^−8^ was applied to identify statistically significant trait associations.

### Multidimensional validation through proteome-wide MR, colocalization, and SMR-HEIDI analyses

To enhance the robustness of genetically supported inferences, we integrated two-sample MR with Bayesian colocalization analysis—two complementary approaches that together provide stronger evidence than that when either method is used alone. MR tests the directional hypothesis that genetically predicted plasma protein levels influence AMD risk, thereby offering initial statistical support for causality. Colocalization extends this by assessing whether the protein and AMD share the same genetically supported variant at a given locus, thereby helping exclude confounding due to LD or the influence of nearby genes. Consistent results from both approaches increase confidence not only in the direction of the effect but also in the shared biological origin of the associations, providing a more specific and reliable basis for inferring genetically supported relationships[26]. Accordingly, we applied this integrative framework to prioritize genetically supported candidate proteins for AMD.

A two-sample MR analysis was performed, treating plasma protein levels as exposures and the previously derived AMD GWAS meta-analysis summary statistics as the outcome. MR analysis for each protein was conducted individually using the TwoSampleMR package in R (https://mrcieu.github.io/TwoSampleMR), applying either the Wald ratio or IVW method as appropriate. A valid MR analysis must satisfy three key assumptions [27]: (1) the IV is associated with the exposure; (2) the IV influences the outcome only through the exposure; and (3) the IV is independent of confounders. We evaluated these assumptions through multiple sensitivity analyses. Heterogeneity was assessed using Cochran’s Q test, whereas directional pleiotropy was examined via MR-Egger regression for proteins with three or more instrumental variants. Instrument strength was quantified using F-statistics, and causal directionality verified through MR Steiger tests. Significant associations were defined as those passing false discovery rate (FDR) correction at 0.05 using the Benjamini–Hochberg method. For significant MR findings, we performed Bayesian colocalization analysis using the coloc R package (https://github.com/chr1swallace/coloc) to determine whether the protein and AMD associations shared a common causal variant. The colocalization analysis evaluates five hypotheses (with posterior probabilities [PPs]): (H0) association with neither trait; (H1) association with trait 1 (protein) only; (H2) association with trait 2 (AMD) only; (H3) association with both traits but driven by two distinct causal variants; and (H4) association with both traits driven by a single shared causal variant. This study primarily focused on the PP for hypothesis 4 (PP.H4), which indicates a shared genetic variant underlying both the protein and AMD. Metabolites exhibiting evidence of a shared genetically supported variant (PP.H4 > 0.5) were considered to have statistically significant MR associations. In the colocalization analysis, we used the default prior probabilities implemented in the coloc R package (p1 = 1e−4, p2 = 1e−4, p12 = 1e−5), which are widely adopted in the field for hypothesis-agnostic genetic colocalization. A threshold of PP.H4 > 0.5 was selected to indicate suggestive evidence of a shared causal variant, following established precedent in the literature. Yuan et al. employed a similar threshold (PP.H4 > 0.5) to define moderate colocalization evidence alongside a stricter tier for high-confidence findings [28]. Our approach adopted this tiered framework to balance sensitivity and specificity in prioritizing genetically supported candidate proteins.

We applied summary-data-based MR (SMR) with the Heterogeneity in Dependent Instruments (HEIDI) test to validate the genes identified through MR and colocalization analyses. Unlike other integrative approaches, SMR combined with HEIDI can differentiate between pleiotropy and linkage models [29]. The SMR method estimated the effect of exposure on AMD by using a two-stage least-squares framework that included exposure-associated genetic variants. The subsequent HEIDI test was used to distinguish between two scenarios: (1) a pleiotropic model, in which a single causal variant influences both the exposure (e.g., protein abundance) and outcome (AMD)—a scenario consistent with shared genetic etiology; and (2) a linkage model, in which two distinct but correlated causal variants independently affect the exposure and outcome, respectively—a scenario that could produce false-positive associations due to LD. By testing for heterogeneity in the SMR estimates across multiple instruments, the HEIDI test helps determine whether the observed association is driven by a shared causal variant rather than by distinct but correlated variants [30]. Associations were considered significant at an SMR *P* value < 0.05 (indicating a statistically significant association between the molecular trait and AMD) and a HEIDI *P* value > 0.05 (indicating no significant heterogeneity, thereby supporting a shared genetic basis and precluding a linkage model). By consistently applying these thresholds across three molecular levels—protein abundance (using pQTL data), gene expression (using eQTL data), and DNA methylation (using mQTL data)—we sought to identify genes with convergent evidence across multiple regulatory layers. Specifically, genes that passed both the SMR and HEIDI thresholds at the protein, transcript, and methylation levels were considered to have the strongest multi-omics support, whereas those with evidence at one or two levels were prioritized accordingly.

### Two-step MR mediation analysis

To investigate the potential mediation of immune traits in the protein–AMD relationship, we implemented a two-step MR approach. First, we performed two-sample MR to identify immune traits genetically associated with AMD, estimating effect β_2_. Second, we applied two-sample MR to estimate the effect of target proteins on these immune traits (β_1_). The total effect (β) of each protein on AMD was estimated separately. Immune traits significantly associated (*P* < 0.05) in both steps were considered putative mediators. The mediation effect was calculated as β_2_ × β_1_, and the proportion mediated as (β_2_ × β_1_)/β. The robustness of the mediation findings was evaluated using previously described sensitivity analyses (Additional file 1: Table S2).

### Functional annotation and disease enrichment analysis

We performed gene ontology (GO) enrichment analysis to functionally characterize the newly identified candidate genes from the GWAS meta-analysis (as listed in Table 1) and the MR-derived protein-coding genes [31]. This analysis identified significantly overrepresented terms across biological process (BP), cellular component (CC), and molecular function (MF) domains, with functional categories considered enriched at *P* < 0.05. We conducted disease enrichment analysis using Metascape [32], which integrates multiple databases, including DisGeNET and OMIM, to evaluate the associations between these genes and human diseases.

**Table 1:** Genome-wide significant loci identified in the meta-analysis of age-related macular degeneration

rsID	Chr	Position	Nearest gene	PoPS gene	EA	NEA	Beta	SE	*P* value
*Candidate variants*									
rs12141142	1	198576887	*ATP6V1G3*	*PTPRC*	C	G	0.202	0.032	2.20e−10
rs148136314	1	197495597	*DENND1B*	*DENND1B*	T	C	− 0.300	0.041	2.42e−13
rs200085570	1	195801887	*KCNT2*	*KCNT2*	T	C	− 0.162	0.023	2.51e−12
rs10033900	4	109737911	*CFI*	*CFI*	T	C	0.058	0.009	1.28e−09
rs11134780	5	157510334	*ADAM19*	*HAVCR2*	T	C	− 0.062	0.011	9.15e−09
rs3801281	7	105249126	*SRPK2*	*KMT2E*	T	C	0.064	0.010	3.30e−10
rs75535593	10	122277685	*BTBD16*	*PLEKHA1*	T	C	0.243	0.030	2.36e−16
rs36114247	13	31237551	*B3GALTL*	*B3GALTL*	T	C	0.068	0.010	1.01e−11
rs11630478	15	74810582	*LMAN1L*	*EDC3*	T	G	0.060	0.010	1.87e−09
rs7289865	22	32695826	*TIMP3*	*FBXO7*	A	C	0.093	0.014	7.55e−11

*Chr* = chromosome; *PoPS* = polygenic priority score; *NEA* = non-effect allele; *EA* = effect allele; *SE* = standard error

Results were determined using a fixed-effect inverse variance-weighted meta-analysis. Chromosome positions are based on the GRCh37/hg19 reference. Note: rs75535593 (10q26, *PLEKHA1/ARMS2/HTRA1* region), rs10033900 (*CFI* region), and rs7289865 (*TIMP3* region) are located within or adjacent to previously reported age-related macular degeneration-associated loci

### scRNA-seq analysis of cell-type-specific enrichment

We analyzed scRNA-seq data from human retina and retinal pigment epithelium (RPE)/choroid tissues (GSE203499) to evaluate the cell-type-specific expression of GWAS and MR/colocalization candidate genes. The data were processed using Seurat [33], and cells expressing > 200 unique genes with < 10% mitochondrial gene counts were retained. Genes detected in < 3 cells were excluded from the analysis. Expression values were normalized using the NormalizeData function and scaled via ScaleData. Cell types were annotated with SingleR [34]. Differential expression between each cell type and all others was assessed using the Wilcoxon rank-sum test. Genes with an average log_2_fold change (log_2_FC) > 0.5 and FDR-adjusted *P* value < 0.05 were considered significantly enriched in a given cell type.

Cell types were annotated using SingleR with reference datasets specific to retinal and choroidal tissues, where available. For clusters that could not be confidently assigned to established retinal cell types (e.g., rod photoreceptors, cone photoreceptors, bipolar cells, ganglion cells, amacrine cells, horizontal cells, Müller glia, RPE, microglia/macrophages, vascular endothelial cells, and pericytes), we employed a conservative approach, specifically labeling them by their most highly expressed marker genes to prioritize transcriptomic accuracy.

Initial cell annotation was performed using an automated reference-based pipeline. However, as such automated algorithms can sometimes assign broad labels that lack tissue-specific resolution, cluster annotations were subsequently verified and refined based on the expression of established functional markers. Notably, a cluster initially flagged by the automated pipeline as “tissue stem cells” exhibited strong enrichment of stromal and mesenchymal-associated genes (e.g., *IGFBP6*, *LAMC2*, and *RBP1*). To accurately reflect the biological identity of this cluster within the retinal microenvironment, it was manually re-annotated as “stromal/progenitor-like cells.”

### Drug target prioritization and safety evaluation

We evaluated candidate genes from candidate GWAS hits and MR/colocalization analyses using the Mouse Genome Informatics (MGI) database (http://www.informatics.jax.org/), which utilizes standardized nomenclature and controlled vocabularies, including the mouse developmental anatomy, mammalian phenotype, and gene ontologies. Among the queried genes, we systematically identified knockout (KO) models exhibiting visual system abnormalities. To assess druggability, we submitted the target genes to the Drug–Gene Interaction Database (DGIdb; http://dsigdb.tanlab.org/DSigDBv1.0/) to classify known or potential drug interactions. We utilized the Enrichr platform (https://maayanlab.cloud/Enrichr/) and Drug Signatures Database (DSigDB) resource, which contains 22,527 gene sets and 17,389 compounds, to predict therapeutic chemicals. Additionally, currently approved AMD drugs and their targets were compiled from the DrugBank database (https://go.drugbank.com).

## Results

**Fig. 1:**
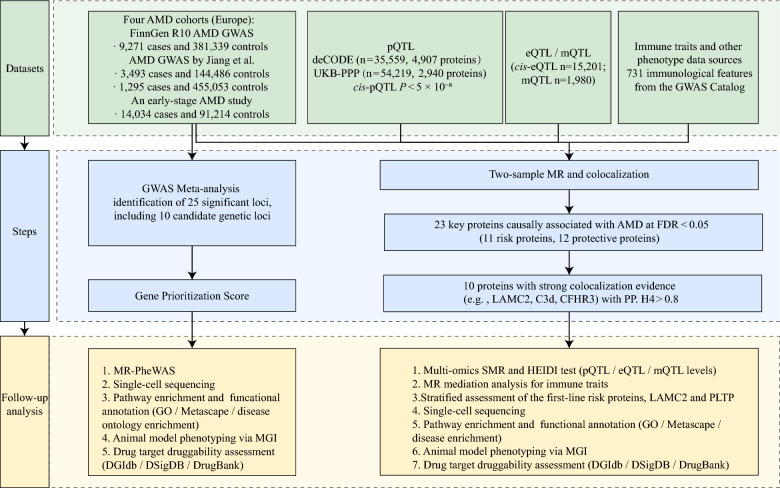
Workflow for identifying and prioritizing therapeutic targets for age-related macular degeneration (AMD). We performed a genome-wide association study (GWAS) meta-analysis to discover candidate loci and conducted proteome-wide Mendelian randomization (MR)/colocalization to identify genetically supported proteins. Key candidates, including the first-tier genes, *LAMC2* and *PLTP*, were subsequently validated through multi-omics integration, immune mediation analysis, and single-cell RNA-seq, culminating in the assessment of their druggability and associated therapeutic compounds. DGIdb, Drug–Gene Interaction Database; DSigDB, Drug Signatures Database; eQTL, expression quantitative trail loci; FDR, false discovery rate; GO, gene ontology; HEIDI, heterogeneity in dependent instruments; MGI, Mouse Genome Informatics; mQTL, methylation quantitative trail loci; MR-PheWAS, Mendelian randomization phenome-wide association study; PP.H4, posterior probability for hypothesis 4; pQTL, protein quantitative trail loci; SMR, summary-data-based Mendelian randomization; UKB-PPP, UK Biobank Pharma Proteomics Project

The overall study design and analytical pipeline are presented in Fig. 1.

### GWAS meta-analysis identifies 10 candidate AMD loci

**Fig. 2:**
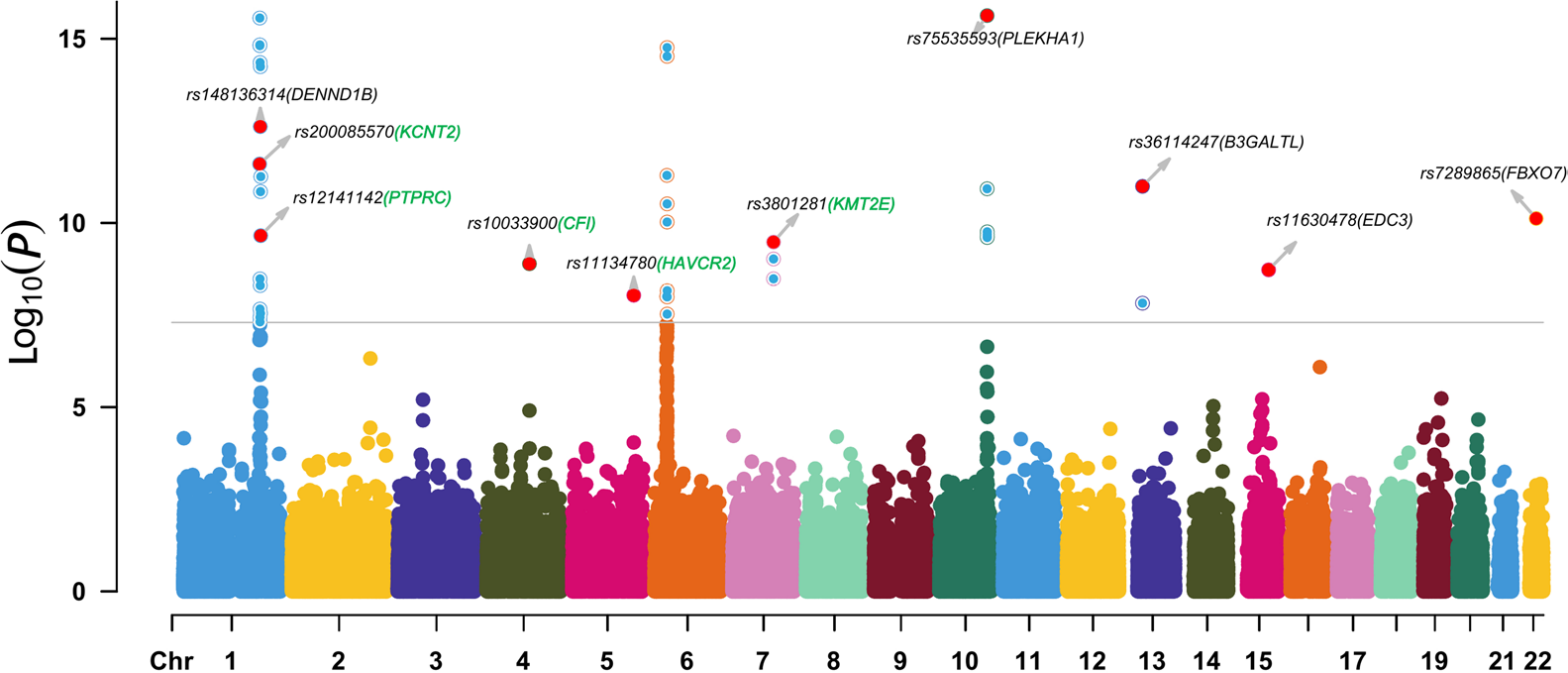
Manhattan plots showing associations with age-related macular degeneration from a genome-wide association study meta-analysis. Red markers represent seven newly discovered variants and target genes identified by polygenic priority scores. Green highlights indicate druggable target gene loci, and the gray line represents the threshold of 5 × 10^−8^

Our meta-analysis of four GWAS datasets (28,543 cases and 1,072,092 controls) identified a total of 25 independent genome-wide significant loci (*P* < 5 × 10^−8^), as shown in Fig. 2. Among these loci, 15 corresponded to or were located within 500 kb of index variants previously reported in the four individual GWAS cohorts included in our meta-analysis. Full details of these loci are provided in Additional file 1: Table S3, demonstrating the robustness of our genetic signals. To prioritize the discovery of candidate therapeutic targets, we focused our downstream functional investigations on a curated subset of the remaining 10 prioritized candidate loci (Table 1). Notably, seven of these represent newly identified candidate associations within our meta-analysis, demonstrating absolute effect sizes (β) ranging from 0.060 to 0.300. The remaining three loci (near *PLEKHA1*/*ARMS2*/*HTRA1*, *CFI*, and *TIMP3*) resided within previously identified AMD-associated regions [35–37]; however, they were retained in this prioritized candidate list as they served as critical variants for our subsequent multi-omics integration and downstream functional analyses. The genomic inflation factor (λ = 1.052) confirms the absence of systematic bias. Furthermore, formal COJO demonstrated that signals at the seven newly identified loci remained robustly significant at the genome-wide level (conditional *P* < 5 × 10^−8^) even after conditioning on previously reported AMD index variants (Additional file 1: Table S4). This finding confirmed the seven newly identified loci as independent genomic loci for AMD, effectively ruling out the possibility that these signals were merely long-range LD extensions of known variants.

To further evaluate the phenotypic heterogeneity, we directly assessed the genetic consistency across cohorts. At the genome-wide level, the LDSC analysis yielded a highly significant genetic correlation (r_g_ = 0.62, *P* = 0.005; Additional file 1: Table S5) between our meta-analysis and the independent advanced AMD cohort. Furthermore, a stratified look-up validation for our 10 lead variants (including seven novel candidate and three previously reported loci) demonstrated 100% concordance in the effect directions within the advanced AMD cohort (Additional file 1: Table S6). These results provide compelling multilayered evidence for a shared genetic basis across AMD stages, robustly justifying the integration of diverse phenotypes.

**Fig. 3:**
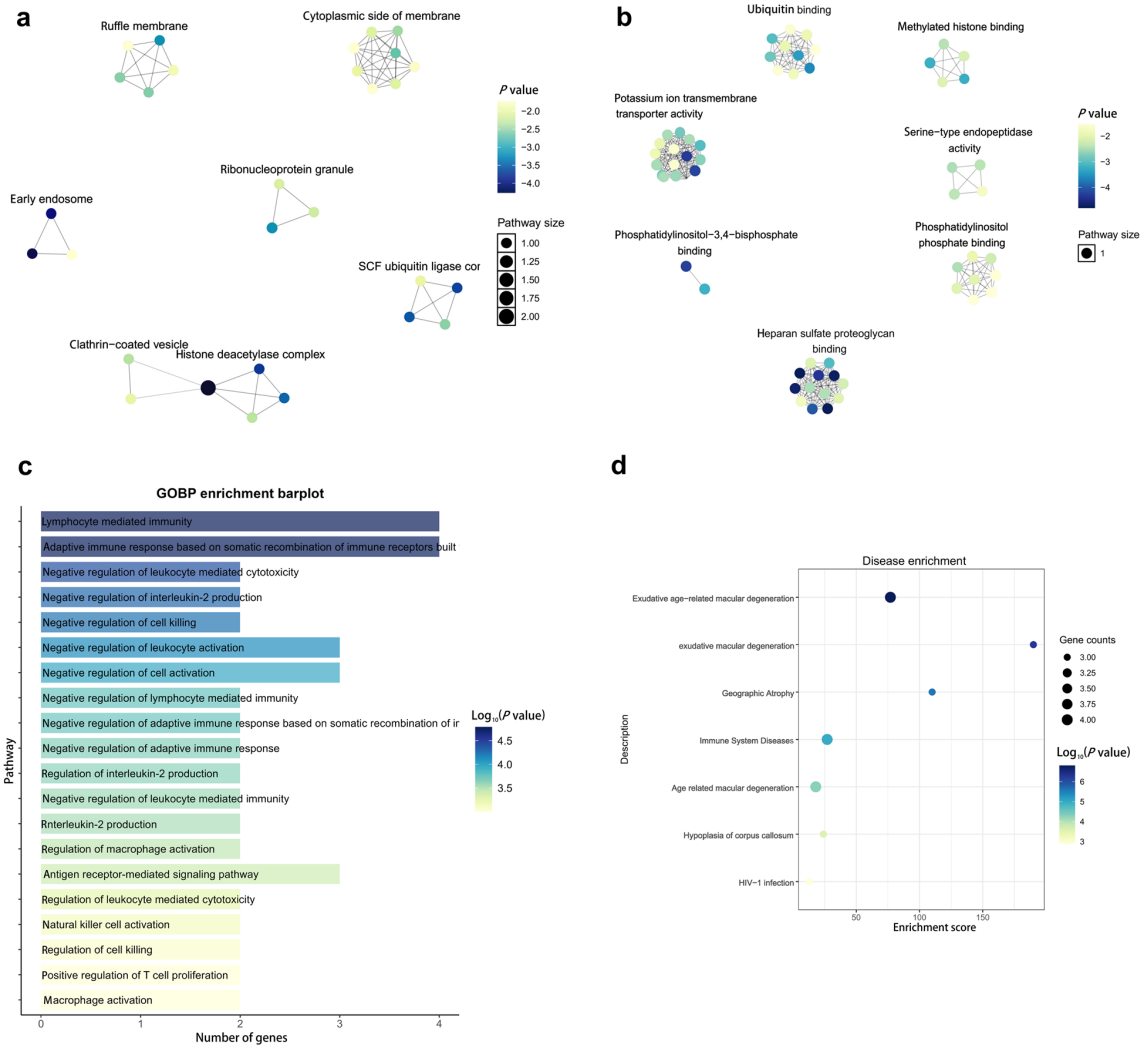
Enrichment analysis of target genes identified from the meta-analysis. **a** Cellular components identified from gene ontology (GO) analysis. **b** Molecular functions identified from GO analysis. **c** Biological processes identified from GO analysis. **d** Enrichment of target genes in disease pathways. GOBP, gene ontology biological processes

The analysis included 10,491,749 SNPs with a MAF of at least 0.01. For each variant, the most likely causal gene was prioritized using the PoPS score, a method that integrates genome-wide features, whereas the nearest gene served as a complementary local annotation, as described in the Methods. GO enrichment analysis was subsequently performed on the 10 identified candidate genes (detailed in Table 1), revealing significant enrichment in specific BPs, such as lymphocyte-mediated immunity, CCs including transcriptionally active chromatin and nuclear speck, and MFs such as phosphatidylinositol phosphate binding (Fig. 3). Standard GO enrichment analysis typically requires multiple genes within a term to achieve statistical significance, which may underrepresent genes with strong biological relevance that do not co-aggregate into enriched categories. Therefore, beyond the aggregated enrichment results, we also prioritized individual genes with robust multi-omics support (e.g., *LAMC2* and *PLTP*) through orthogonal approaches, including proteome-wide MR, colocalization analysis, SMR analysis across multiple molecular layers, and single-cell expression profiling, ensuring that potentially important single-gene findings were not overlooked. Disease enrichment analysis further associated these genes with conditions that included neovascular AMD, geographic atrophy, and immune system diseases.

### MR-PheWAS analysis of the 10 candidate AMD loci

To provide a broader systemic context and preliminary safety insights for the identified genetic variants, we performed a supplementary exploratory MR-PheWAS (Additional file 1: Table S7). Except for rs200085570/KCNT2, all other candidate AMD variants were significantly associated with at least one non-AMD-related disease or trait.

MR-PheWAS revealed distinct pleiotropic patterns. The rs10033900/CFI variant regulates the complement system, and the rs11630478/EDC3 variant is associated with cardiometabolic traits, including diastolic and systolic blood pressure, hypertension, low-density lipoprotein cholesterol, red blood cell count, and platelet distribution width. The rs12141142/PTPRC variant, which influences lymphocyte count, may contribute to AMD via immune regulation. The rs148136314/DENND1B variant is linked to coagulation Factor XIII, suggesting a role in coagulation pathways, and the rs3801281/KMT2E variant is associated with sun exposure and vitamin D traits, such as sunburn, skin pigmentation, and 25-hydroxyvitamin D levels. The rs11134780/HAVCR2 variant is associated with lung function (FEV1/FVC, peak expiratory flow), HbA1c, and platelet parameters. The rs7289865 variant correlated with anthropometry: height, trunk fat-free mass, and childhood body height. These associations align with established mechanisms of AMD pathogenesis, encompassing complement activation, cardiometabolic dysregulation, immune responses, and ultraviolet exposure. While exploratory, these findings provide a complementary systemic context, highlighting the complex multi-pathway nature of AMD and offering a foundational perspective for evaluating the broader safety profiles of these genetic targets.

### Proteome-wide MR and colocalization analyses identify 23 key proteins

We employed *cis*-pQTLs derived from the deCODE dataset as the primary discovery dataset (assessing 1,812 plasma proteins), and used *cis*-pQTLs from the UKB-PPP dataset as an independent replication source (assessing 1114 plasma proteins). To balance discovery sensitivity with replication rigor, we adopted a two-stage strategy: proteins significantly associated with AMD in deCODE (FDR < 5%) were further subjected to genetic colocalization analysis; proteins with evidence of a shared genetically supported variant (PP.H4 > 0.5) in deCODE were subsequently examined in UKB-PPP for consistency in the direction of the effect. A protein was considered to have suggestive evidence of an association if it met both criteria—significant MR and colocalization support in deCODE and directionally consistent estimates in UKB-PPP, acknowledging that differences in protein coverage between platforms may preclude replication for some proteins.

**Fig. 4:**
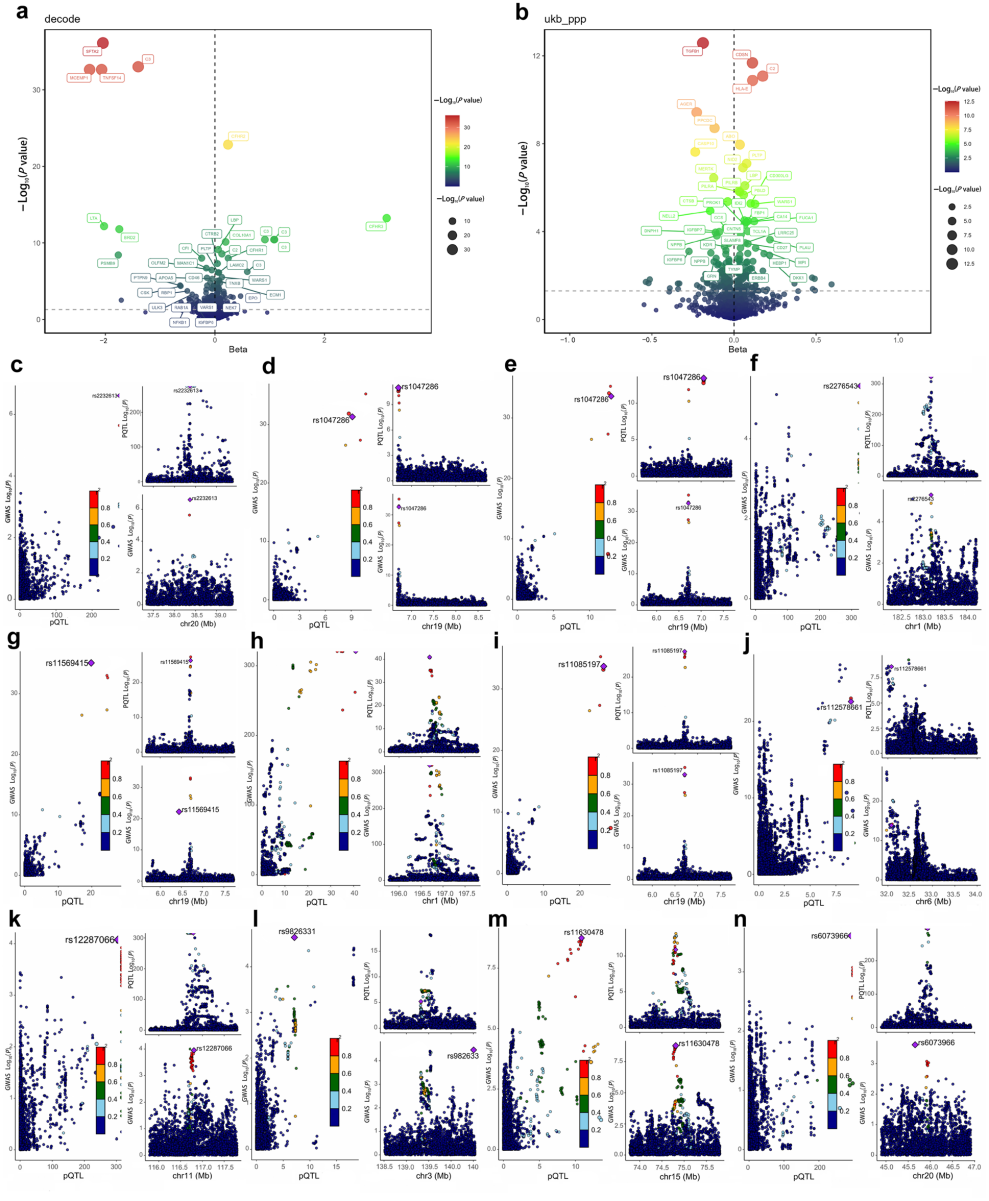
Mendelian randomization (MR) and colocalization analysis results for the full proteome. **a** Volcano plot of the MR analysis using deCODE protein quantitative trait loci (pQTL) data. **b** MR results using UK Biobank Pharma Proteomics Project (UKB-PPP) pQTL data. **c**–**n** Colocalization results for the proteins LBP, MCEMP1, TNFSF14, LAMC2, C3d, CFHR3, C3b, BRD2, APOA5, RBP1, CSK, and PLTP, highlighting their potential associations with age-related macular degeneration

In the deCODE dataset, 89 proteins were significantly associated with AMD (Fig. 4a, Additional file 1: Table S8A). Colocalization analysis refined these to 12 proteins with evidence of a shared genetically supported variant (PP.H4 > 0.5), including 10 with strong evidence (PP.H4 > 0.8): LBP, MCEMP1, TNFSF14, LAMC2, C3d, CFHR3, C3b, BRD2, APOA5, and RBP1 (Fig. 4c–n, Additional file 1: Table S9). Replication in the UKB-PPP dataset confirmed that 13 proteins were significantly associated with AMD in both studies (Fig. 4b, Additional file 8b). Differences in the number of measured proteins between platforms precluded the replication of some deCODE findings.

**Fig. 5:**
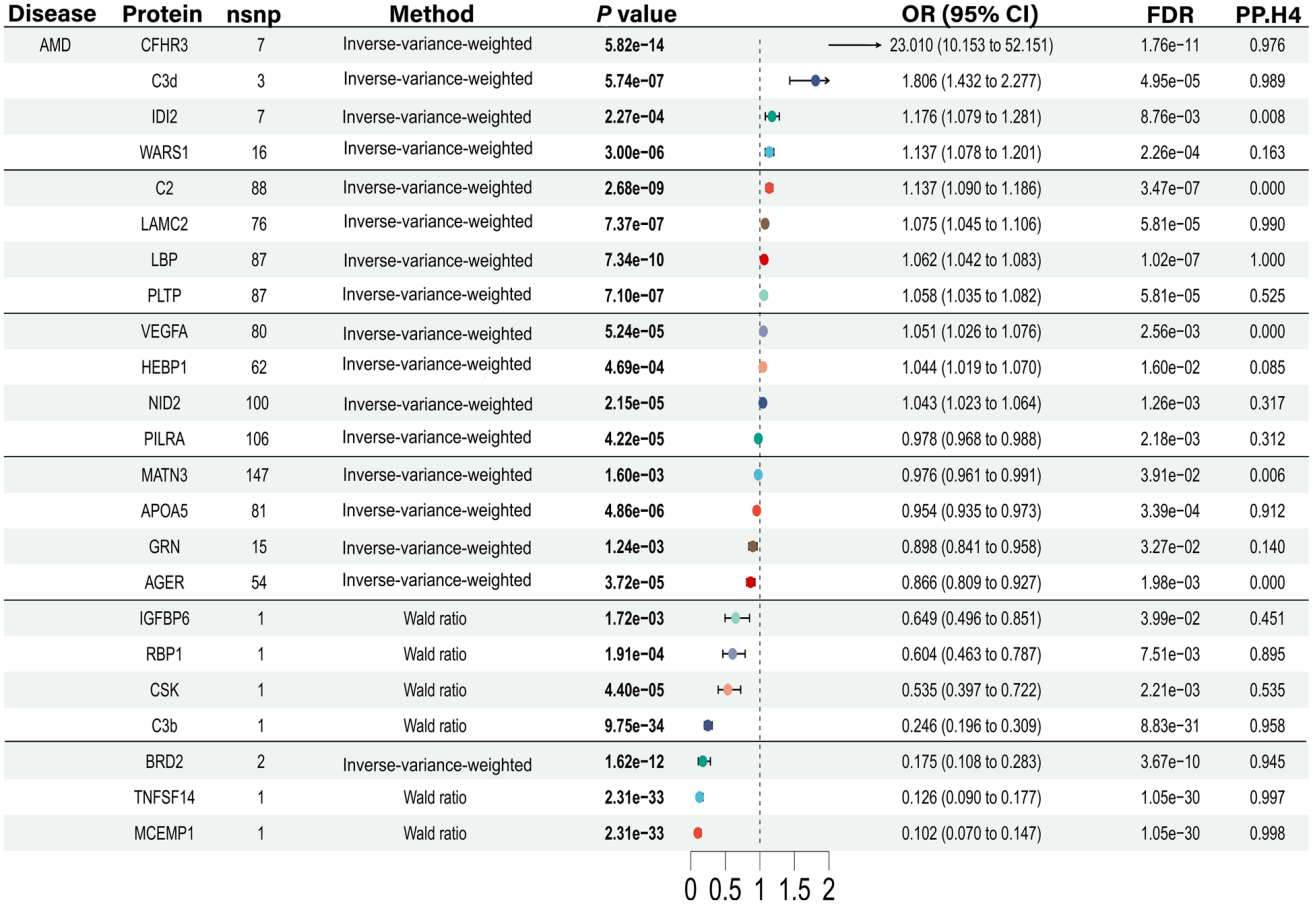
Forest plot of Mendelian randomization (MR) results for the full proteome analysis. The plot displays the odds ratios (ORs) for proteins associated with age-related macular degeneration (AMD), with their corresponding 95% confidence intervals (CIs). Posterior probability for hypothesis 4 (PP.H4) indicates the likelihood that a shared genetic variant is genetically supported for both protein expression and AMD. Results with a false discovery rate (FDR) < 0.05 were considered statistically significant. nsnp indicates the number of instrumental variables used in the MR analysis, namely the number of SNPs serving as genetic instruments

After applying this two-stage discovery–replication framework, we identified 23 proteins with suggestive evidence of association with AMD (Fig. 5), including 11 potential risk and 12 potentially protective factors. Among these, 13 proteins were measured in both datasets and showed a consistent direction of effect; the remaining 10 were uniquely measured in deCODE but met the stringent MR and colocalization thresholds and were thus presented as candidates for further validation.

Importantly, these inferred genetically supported relationships reflect systemic protein influences based on plasma measurements. Further studies integrating ocular tissue proteomics are required to confirm local intraocular dynamics. Sensitivity analyses using multiple methods, such as MR-Egger, weighted median, MR-PRESSO, and contamination mixture, showed minimal horizontal pleiotropy (Additional file 1: Table S8A, B). Genetic instruments for each protein exhibited strong instrument strength (F-statistics; Additional file 1: Table S8C, D), and Steiger directionality tests and filtering supported a genetic direction from protein to AMD without evidence of reverse causality (Additional file 1: Tables S10, S11).

### Multi-tissue transcriptome-wide SMR analysis for AMD

**Fig. 6:**
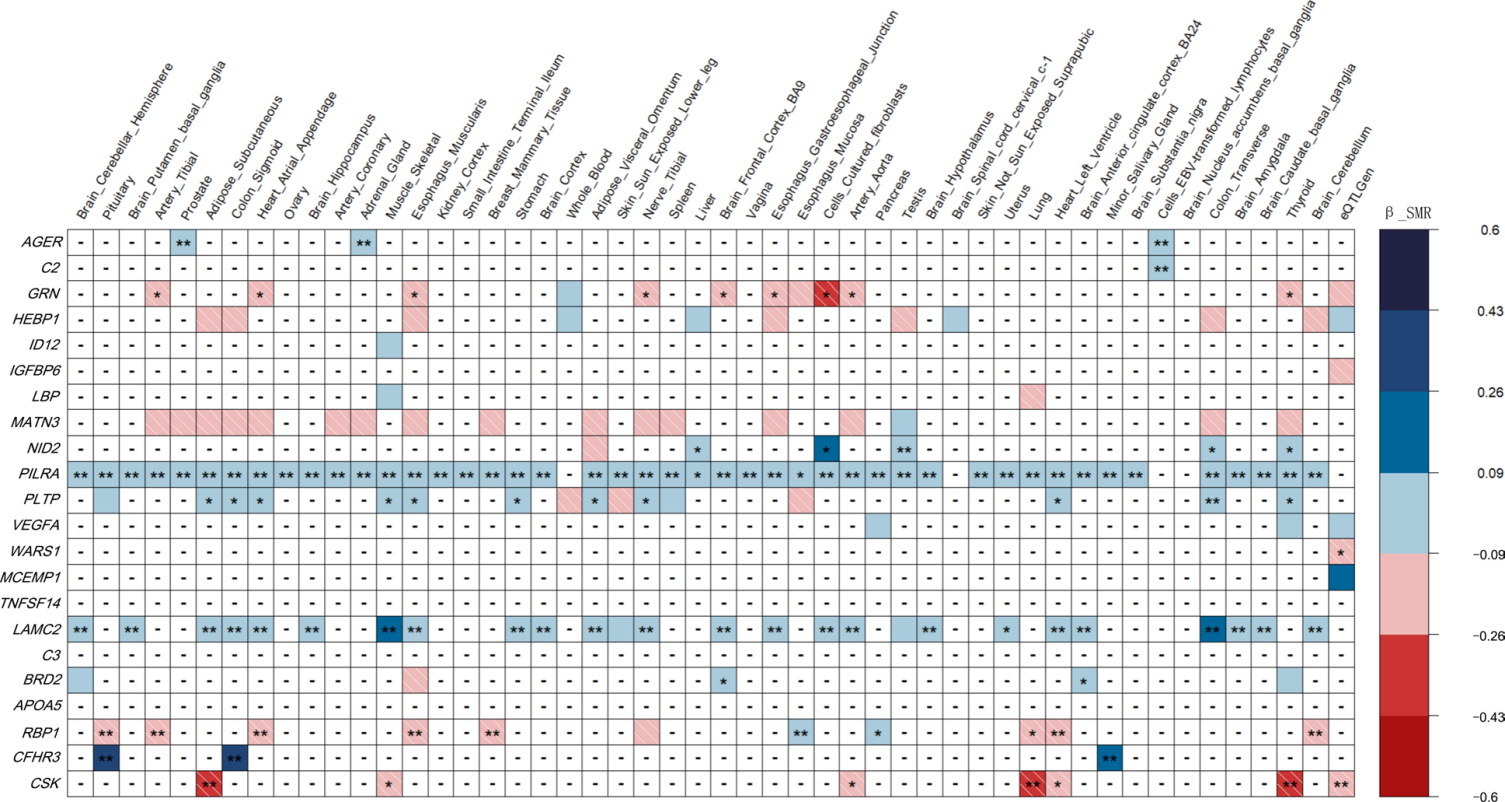
Heatmap of the identified protein-coding genes associated with age-related macular degeneration (AMD). The heatmap shows the effect of plasma and tissue-specific protein-coding gene expression on AMD risk for the identified proteins. Colors represent the β estimates from the summary-data-based Mendelian randomization (SMR) analysis, with red indicating a decrease in AMD risk and blue an increase in AMD risk with each standard deviation increase in gene expression. *P < 0.05; **multiple testing, *P* < 0.05/22 (genes). Values marked with “-” represent missing data due to the lack of valid expression quantitative trait loci in the SMR analysis or failure in the heterogeneity in dependent instruments test

We mapped the 23 key proteins to 22 protein-coding genes. To examine their roles at the transcriptomic level, we performed a large-scale SMR analysis across 49 tissues from the GTEx project and eQTLGen consortium, comprehensively assessing tissue-specific associations (Additional file 1: Table S12). SMR analysis results for the 22 genes are presented in Fig. 6. Significance was defined as an SMR *P* value < 0.05 and HEIDI *P* value > 0.05.

Our SMR analysis validated the associations of 12 protein-coding genes across multiple blood- or tissue-specific tissues. Increased expression of *AGER*, *C2*, *NID2*, *PILRA*, *PLTP*, *LAMC2*, *BRD2*, and *CFHR3* was associated with a higher AMD risk, with *C2*, *NID2*, *PLTP*, *LAMC2*, and *CFHR3* showing consistent direction with the proteomic MR results. *PILRA* expression was associated with AMD risk in 45 tissues, most significantly in the esophagus muscularis (*P*_SMR = 7.5322 × 10⁻^5^, β_SMR = 0.144). *LAMC2* expression was associated with risk in 24 tissues, most strongly in tibial nerve (*P*_SMR = 7.47 × 10^−6^, β_SMR = 0.045). Conversely, elevated expression of *GRN*, *WARS1*, *RBP1*, and *CSK* was associated with reduced AMD risk, with *GRN*, *RBP1*, and *CSK* expression being consistent with the proteomic MR findings. *RBP1* expression was significantly protective in eight tissues, most notably in tibial artery.

Some genes exhibited context-specific effects. For example, although *RBP1* expression in the tibial artery was protective, its expression in the esophageal mucosa was linked to an increased risk. These results corroborate the involvement of key genes identified via MR in AMD pathogenesis and provide transcriptome-level evidence to inform drug target prioritization. Detailed results of the SMR analysis are provided in Additional file 1: Table S13. Of the 23 proteins analyzed for AMD, 13 (LAMC2, LBP, APOA5, PLTP, PILRA, NID2, AGER, WARS1, IGFBP6, GRN, HEBP1, C2, and MATN3) passed the significance thresholds for both the SMR (*P*_SMR < 0.05) and HEIDI (*P*_HEIDI > 0.05) tests. Notably, for all 13 proteins, the direction of the effect estimated through SMR was consistent with the findings of the primary proteomic MR analysis.

### Epigenome-wide SMR analysis for AMD

We performed an SMR analysis using mQTL data for the 22 key protein-coding genes, which identified 23 CpG sites significantly associated with AMD at a transcriptome-wide significance level (*P*_SMR < 0.05, *P*_HEIDI > 0.05; Table 2). These CpG sites were mapped to seven AMD-associated genes. Notably, several CpG sites were associated with an increased AMD risk. These included cg01417625 near *LAMC2* (β_SMR = 0.105), cg10843707 (β_SMR = 0.035) and cg26249834 (β_SMR = 0.098) near *NID2*, and cg21098005 near *PLTP* (β_SMR = 0.115). This finding is consistent with our previous analyses, which identified LAMC2, NID2, and PLTP as pathogenic factors for AMD at both the protein and gene expression levels. The most significant association was observed for cg05284873 near *C3*, which was associated with a reduced AMD risk (β_SMR = − 0.235, *P*_SMR = 1.28 × 10^−8^).

**Table 2:** Summary-data-based Mendelian randomization analysis of methylation quantitative trait loci data for 22 key coding genes

Gene	Probe ID	CHR	BP	β_SMR	*P*_SMR	*P*_HEIDI
*C3*	cg05284873	19	6709822	− 0.235	1.28e−08	0.200
*LAMC2*	cg06211724	1	183155437	− 0.134	3.39e−05	0.872
	cg04218899	1	183155366	− 0.131	3.18e−05	0.768
	cg23696949	1	183155644	− 0.071	8.93e−06	0.963
	cg05929019	1	183155154	− 0.129	3.11e−05	0.893
	cg01949993	1	183155116	− 0.159fd	6.52e−05	0.835
	cg01417625	1	183187433	0.105	4.82e−02	0.541
	cg22346461	1	183155573	− 0.107	9.96e−04	0.300
*RBP1*	cg12298268	3	139257866	0.167	1.28E−03	0.460
	cg15288618	3	139257775	0.156	1.02e−03	0.142
	cg06726820	3	139259742	0.033	3.90e−04	0.103
	cg08972081	3	139259435	− 0.094	5.22e−04	0.077
*BRD2*	cg26246658	6	32936741	− 0.115	2.01E−02	0.524
	cg26595909	6	32946524	0.150	6.31e−05	0.081
	cg02448295	6	32941126	0.049	2.57e−03	0.079
	cg07954885	6	32935263	− 0.102	1.88e−02	0.138
	cg08491668	6	32935236	− 0.063	1.57e−02	0.072
*NID2*	cg25277187	14	52535028	− 0.070	1.82e−03	0.480
	cg10843707	14	52510701	0.035	5.61e−03	0.283
	cg26249834	14	52479816	0.098	3.26e−03	0.085
*IGFBP6*	cg04791129	12	53490632	− 0.114	2.27e−03	0.557
	cg23906641	12	53496020	− 0.126	6.94e−03	0.296
*PLTP*	cg21098005	20	44538605	0.115	4.14e−02	0.121

The analysis identified 23 CpG sites associated with age-related macular degeneration. Gene names are listed alongside their corresponding probe IDs, chromosome locations (CHR), base-pair positions (BP), summary-data-based Mendelian randomization (SMR) beta estimates (β_SMR), SMR *P* values (*P*_SMR), and HEIDI *P* values (*P*_HEIDI)

### Integration of multi-omics evidence and enrichment analysis for AMD-associated genes

We integrated results from the MR, colocalization, and multi-omics SMR analyses to systematically prioritize candidate genes influencing AMD. As proteins represent functional gene products, all candidates were implicated at the protein level. We classified these genes into five tiers based on predefined molecular evidence (Table 3).

**Table 3:** Mendelian randomization-identified target genes and their druggability assessment

Gene	Protein	MR direction	PP.H4	SMR			Druggable gene
				Passed protein-level analysis (direction)	Passed gene-level analysis (direction)	Passed DNA methylation-based analysis (direction)	
Tier1							
*LAMC2*	LAMC2	+	0.990	Yes (+)	Yes (+)	Yes (+/−)	Yes
*PLTP*	PLTP	+	0.525	Yes (+)	Yes (+)	Yes (+)	Yes
Tier2							
*LBP*	LBP	+	1.000	Yes (+)	−	−	−
*CFHR3*	CFHR3	+	0.976	−	Yes (+)	−	−
*C3*	C3d	+	0.989	−	−	Yes (−)	Yes
	C3b	−	0.958	−	−	Yes (−)	Yes
*BRD2*	BRD2	−	0.945	−	Yes (+)	Yes (+/−)	Yes
*APOA5*	APOA5	−	0.912	Yes (−)	−	−	Yes
*RBP1*	RBP1	−	0.895	−	Yes (−)	Yes (+/−)	−
*CSK*	CSK	−	0.535	−	Yes (−)	−	Yes
Tier3							
*NID2*	NID2	+	0.317	Yes (+)	Yes (+)	Yes (+/−)	−
*MCEMP1*	MCEMP1	−	0.998	−	−	−	−
*TNFSF14*	TNFSF14	−	0.997	−	−	−	Yes
Tier4							
*WARS1*	WARS1	+	0.163	Yes (+)	Yes (−)	−	−
C2	C2	+	1.17e−42	Yes (+)	Yes (+)	−	−
*HEBP1*	HEBP1	+	0.085	Yes (+)	−	−	−
*PILRA*	PILRA	−	0.312	Yes (−)	Yes (+)	−	−
*GRN*	GRN	−	0.140	Yes (−)	Yes (−)	−	−
*AGER*	AGER	−	3.50e−41	Yes (−)	Yes (+)	−	−
*MATN3*	MATN3	−	0.006	Yes (+)	−	−	−
*IGFBP6*	IGFBP6	−	0.451	Yes (−)	−	Yes (−)	Yes
Tier5							
*VEGFA*	VEGFA	+	5.10e−13	−	−	−	Yes
*IDI2*	IDI2	+	0.008	−	−	−	−

This table summarizes results of the Mendelian randomization (MR) analysis for age-related macular degeneration-related target genes and their druggability evaluation. The “MR direction” column indicates the direction of the effect, with “+” representing a positive association and “−” a negative association; the “PP.H4” column shows the posterior probability for hypothesis 4 (PP.H4) obtained from colocalization analysis; the “SMR” column indicates whether the gene passed the summary-data-based MR (SMR) analysis; and the “Druggable gene” column indicates whether the gene is considered druggable. Genes are categorized into five tiers based on reliability of the evidence, with Tier 1 indicating strong evidence and druggability, and Tier 5 indicating weaker evidence

Tier 1: Genes with MR and colocalization support (PP.H4 > 0.5) at the protein level and SMR significance across all three molecular levels—protein abundance, gene expression, and DNA methylation (*P*_SMR < 0.05 and *P*_HEIDI > 0.05 at each level). Tier 2: Genes with MR and colocalization support at the protein level and SMR significance at one or two molecular levels. Tier 3: Genes that passed the MR analysis at the protein-abundance level and met at least one of the following criteria: (1) passed colocalization at the protein level or (2) passed the SMR analysis across all three molecular levels (protein, gene expression, and DNA methylation). Tier 4: Genes with MR significance but no colocalization support (PP.H4 ≤ 0.5) and SMR significance in at least one molecular level. Tier 5: Genes with MR significance but lacking support from both colocalization and SMR analyses. Based on this multi-omics framework, LAMC2 and PLTP were prioritized as Tier 1 risk proteins for AMD, supported by convergent genetic evidence across all analytical layers.

Furthermore, GO analysis revealed BPs and CCs associated with the MR-identified target genes (Additional file 2: Fig. S1). In the BP domain, the target genes were significantly enriched for terms such as the positive regulation of leukocyte migration, chemotaxis, and receptor-mediated endocytosis. The term most significantly enriched in the CC domain was high-density lipoprotein particle. Within the MF domain, the most prominent enrichment was observed for fibronectin binding.

### Mediating role of immune cells in the effect of MR-identified proteins on AMD

**Fig. 7:**
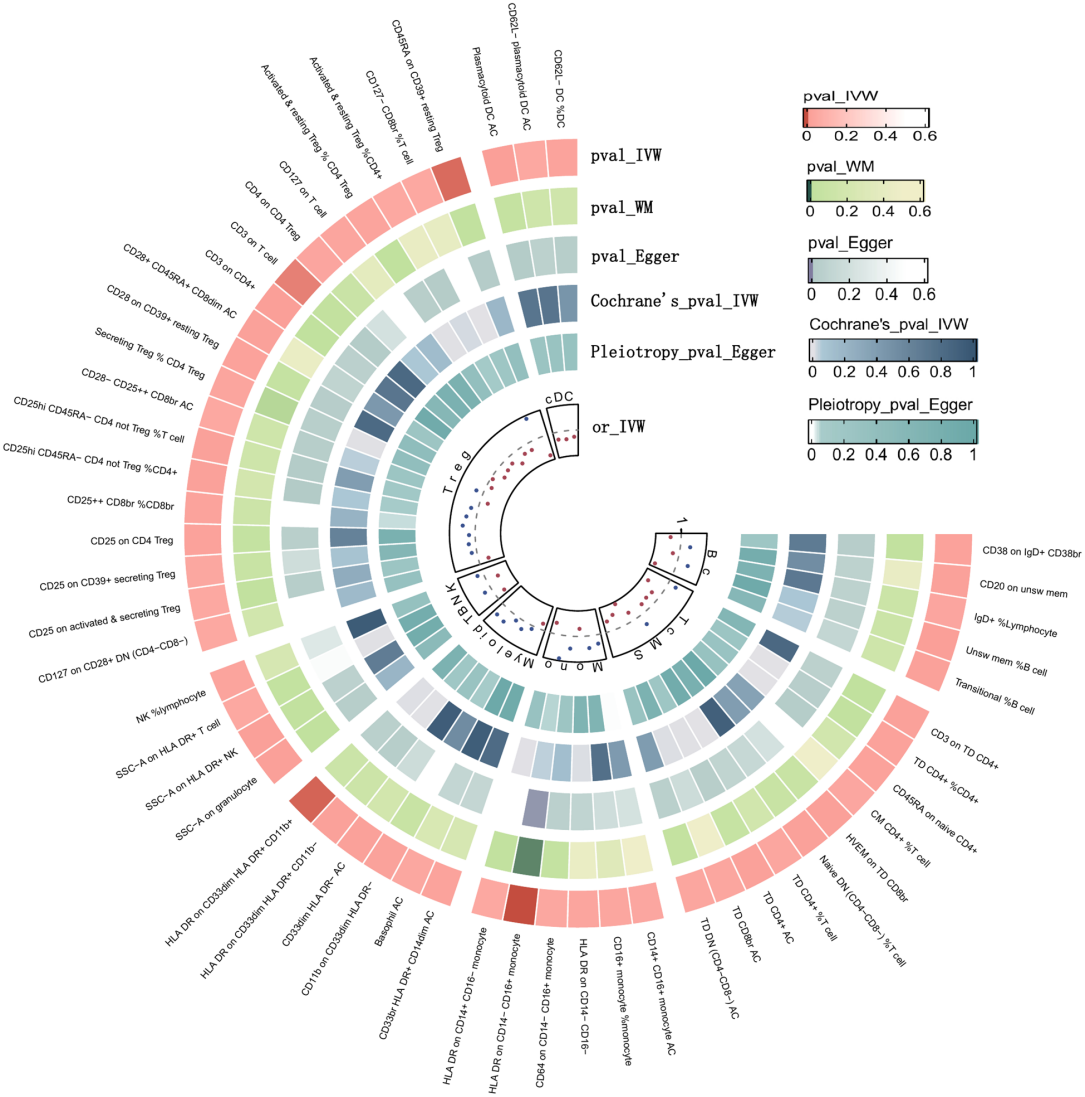
Circular heatmap displaying the Mendelian randomization (MR) analysis results of 731 immune traits and age-related macular degeneration. The circular heatmap shows the *P* values from three MR methods: pval_IVW (inverse-variance weighted), pval_WM (weighted median), and pval_Egger (MR-Egger). The figure also includes heterogeneity and pleiotropy test results, represented by Cochran’s pval_IVW and Pleiotropy_pval_Egger, respectively. Color intensity in the heatmap indicates the strength of the association

Given the significant enrichment of MR-identified genes in immune-related processes, we investigated whether immunological traits mediate the relationship between protein targets and AMD. We initially estimated the effects of 731 immune traits on AMD and identified 53 significantly associated traits, among which regulatory T cell (Treg)-related traits showed the most prominent associations (Fig. 7, Additional file 1: Table S5). We then performed a mediation analysis to estimate the effects of the 23 protein targets on these 53 immune traits, retaining only results where the total and mediation effects were concordant in direction (Table 4).

**Table 4:** Mediation effect analysis of identified proteins on age-related macular degeneration

Protein	Outcome	Mediator	β1	β2	Mediation effect	SE	Total effect	Mediation proportion (%)
LAMC2	AMD	SSC-A on granulocyte (TBNK)	− 0.0607	− 0.0332	0.0020	0.0019	0.0725	2.78
	AMD	CD28^+^ CD45RA^+^ CD8^dim^ AC (Treg)	− 0.0669	− 0.0065	0.0004	0.0018	0.0725	0.60
	AMD	Activated and resting Treg %CD4^+^	− 0.0864	− 0.0256	0.0022	0.0024	0.0725	3.05
	AMD	Activated and resting Treg %CD4 Treg	− 0.0686	− 0.0244	0.0017	0.0019	0.0725	2.30
	AMD	Secreting Treg %CD4 Treg	0.0668	0.0233	0.0016	0.0018	0.0725	2.15
C3d	AMD	Basophil AC (myeloid cell)	0.6800	0.0105	0.0071	0.1824	0.5911	1.20
	AMD	CD33^dim^ HLA DR^-^ AC (myeloid cell)	0.7284	0.0128	0.0093	0.1959	0.5911	1.58
	AMD	Plasmacytoid DC AC (cDC)	− 0.3873	− 0.0243	0.0094	0.0742	0.5911	1.59
PILRA	AMD	HLA DR on CD33^dim^ HLA DR^+^ CD11b^-^ (myeloid cell)	− 0.0500	0.0392	− 0.0020	0.0011	− 0.0223	8.78
	AMD	HLA DR on CD33^dim^ HLA DR^+^ CD11b^+^ (myeloid cell)	− 0.0715	0.0366	− 0.0026	0.0014	− 0.0223	11.69
	AMD	CD25 on CD39^+^ secreting Treg	− 0.0598	0.0219	− 0.0013	0.0008	− 0.0223	5.85
	AMD	CD127 on Treg	0.0271	− 0.0452	− 0.0012	0.0010	− 0.0223	5.49
	AMD	NK %lymphocyte (TBNK)	− 0.0262	0.0200	− 0.0005	0.0004	− 0.0223	2.35
VEGFA	AMD	TD CD4^+^ %T cell (maturation stages of Treg)	− 0.0651	− 0.0298	0.0019	0.0021	0.0493	3.93
	AMD	TD CD4^+^ %CD4^+^ (maturation stages of Treg)	− 0.0679	− 0.0623	0.0042	0.0027	0.0493	8.58
	AMD	Secreting Treg %CD4 Treg	0.0553	0.0233	0.0013	0.0015	0.0493	2.62
	AMD	CD62L^-^ plasmacytoid DC AC (cDC)	− 0.0659	− 0.0274	0.0018	0.0018	0.0493	3.67
CFHR3	AMD	NK %lymphocyte (TBNK)	0.3927	0.0200	0.0079	0.0720	3.1359	0.25

*AMD* = age-related macular degeneration; *SE* = standard error; *SSC* = side scatter area; *Treg* = regulatory T cell

β1 and β2 represent the direct effects of proteins and immune traits on AMD, respectively. The “Mediation effect” column reflects the mediating role of immune traits between proteins and AMD; the “Total effect” column indicates the overall effect of the protein on AMD; and the “Mediation proportion” column represents the percentage of the total effect mediated by immune traits

Exploratory mediation analysis suggested that the effect of LAMC2 on AMD might be partially mediated by several immune-cell populations: side scatter area (SSC)-A on granulocytes (TBNK), CD28^+^ CD45RA^+^ CD8^dim^ AC (Treg), activated and resting Treg %CD4^+^, activated and resting Treg %CD4 Treg, and secreting Treg %CD4 Treg. The modest mediation proportions measured (0.60%–3.05%) suggest that these exploratory findings delineate a partial, hypothesis-generating immune mechanism within a broader pathogenic network.

The effect of C3d on AMD was mediated by basophil AC (myeloid cell), CD33^dim^ HLA DR^-^AC (myeloid cell), and plasmacytoid DC AC (cDC) immune-cell populations, with mediation proportions of 1.20%, 1.58%, and 1.59%, respectively. Furthermore, the effect of PILRA on AMD was mediated by HLA DR on CD33^dim^HLA DR^dim^ CD11b^-^ (myeloid cell), HLA DR on CD33^dim^HLA DR^+^ CD11b^+^ (myeloid cell), CD25 on CD39^+^ secreting Treg, CD127 on T cell (Treg), and natural killer (NK) %lymphocyte (TBNK) immune-cell populations. The mediation proportions for these immune traits were 8.78%, 11.69%, 5.85%, 5.49%, and 2.35%, respectively. The effect of VEGFA was mediated by TD CD4^+^%T cell (maturation stages of T cell), TD CD4^+^ %CD4^+^ (maturation stages of T cell), secreting Treg %CD4 Treg, and CD62L-cDC immune-cell populations, with respective proportions of 3.93%, 8.58%, 2.62%, and 3.67%. Finally, the effect of CFHR3 on AMD was specifically mediated by TBNK, accounting for a mediation proportion of 0.25%.

### *Mouse KO models for candidate genes identified *via* GWAS or MR proteomics*

We investigated the MGI resource for KO mouse models to provide complementary post-experimental evidence supporting the involvement of target genes in AMD pathophysiology, acknowledging that KO models primarily assess loss-of-function effects (Additional file 1: Table S6). For 11 queried genes (three from GWAS: *EDC3*, *KMT2E*, and *PLEKHA1*; eight from MR proteomics: *RBP1*, *CFHR3*, *C3*, *VEGFA*, *GRN*, *CSK, BRD2 and APOA5*), we retrieved evidence of KO models associated with ocular abnormalities. Among these, KO models of *RBP1*, *CFHR3*, *C3*, *VEGFA*, *GRN*, and *CSK* exhibited phenotypes that included abnormal horizontal cell, RPE, and retinal vasculature morphologies, as well as abnormal electroretinography. Although these observations support a functional role for these genes, further models are required to distinguish between specific loss-of-function versus gain-of-function mechanisms. Overall, these findings suggest an intrinsic regulatory role for these genes in AMD pathogenesis.

### Single-cell expression analysis identifies cell-type-specific expression patterns of GWAS and MR candidate genes in retinal tissues of patients with AMD

**Fig. 8:**
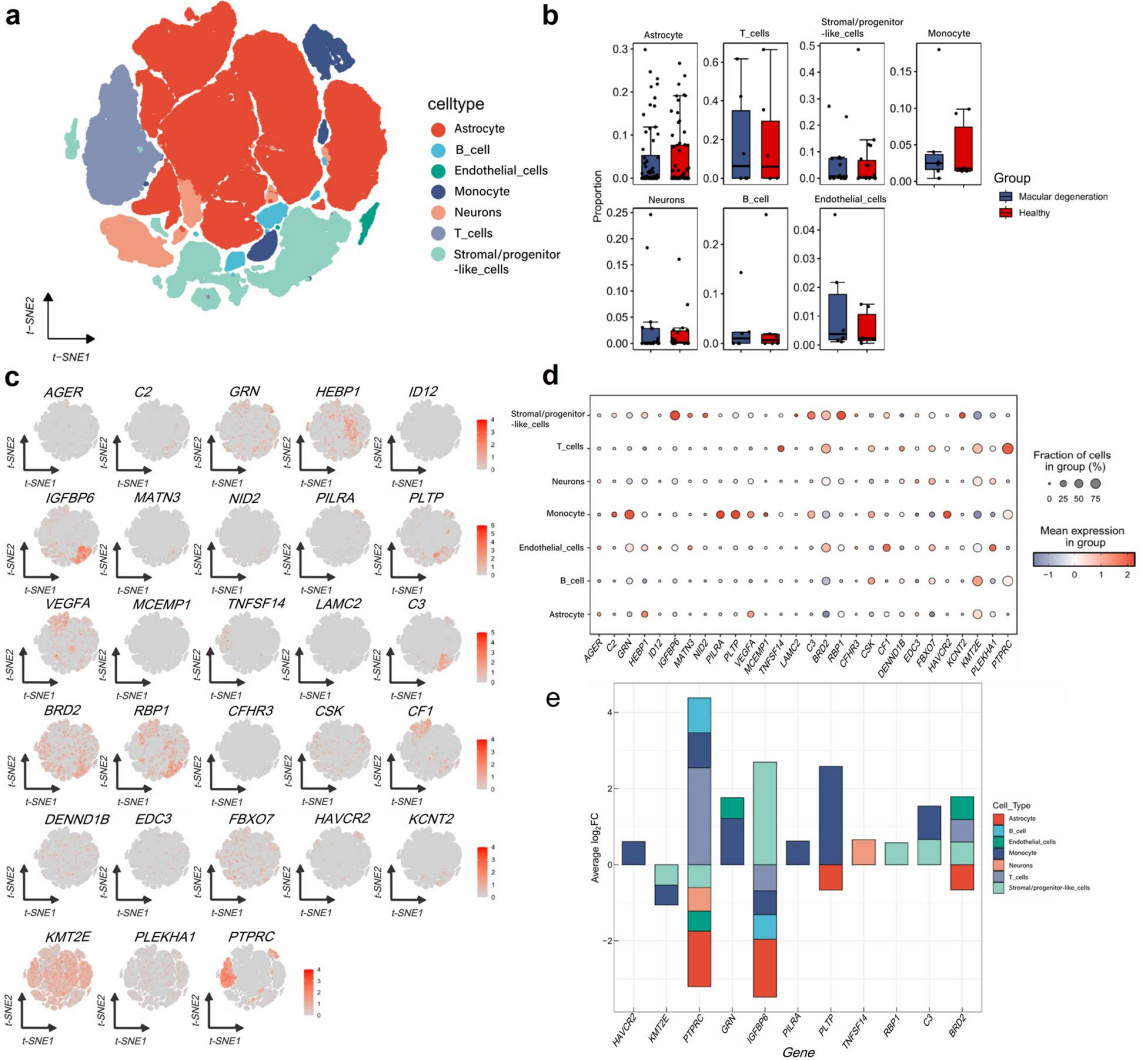
Single-cell expression analysis of 32 target genes identified from the genome-wide association study meta-analysis and Mendelian randomization (MR) in age-related macular degeneration retinal tissue. **a**, **b** Clustering results, with cells grouped into seven major categories based on marker gene expression: astrocytes, B cells, endothelial cells, monocytes, neurons, T cells, and a stromal/progenitor-like population. Due to the dataset resolution, further subdivision of retinal neuronal subtypes was not possible. **c**, **d** Expression of genes in each cluster. **e** Eleven genes had evidence of enrichment in a cell type at an average − log_2_fold change (− log_2_FC) > 0.5 and false discovery rate < 0.05 level

To investigate cell-type-specific enrichment of the 32 target genes prioritized in our GWAS meta-analysis and MR in AMD retinal tissues, we analyzed scRNA-seq data from the Gene Expression Omnibus database. Using a publicly available scRNA-seq dataset (GSE203499) as a discovery resource, we clustered cells into 20 groups and annotated seven major cell categories. These categories include astrocytes, B cells, endothelial cells, monocytes, neuronal clusters, T cells, and a cluster characterized by the expression of genes associated with stromal or progenitor-like states (Fig. 8a). The neuronal clusters comprise multiple retinal neuron subtypes that could not be resolved separately due to limited sequencing depth.

Compared with normal tissues, AMD tissues had lower proportions of astrocytes and monocytes but higher proportions of B cells, endothelial cells, neurons, T cells, and stromal/progenitor-like cells (Fig. 8b). Expression patterns of the 28 coding genes across these clusters are shown in Fig. 8c, d. Among these expression patterns, 11 target genes showed differential expression patterns in specific cell types in AMD retinas (mean log_2_FC > 0.5, FDR-adjusted *P* < 0.05; Fig. 8e, Additional file 1: Table S7). Specifically, *HAVCR2*, *GRN*, *PLTP*, and *C3* were enriched mainly in monocytes; *IGFBP6* and *RBP1* in stromal/progenitor-like cells; and *PTPRC* in T cells, B cells, and monocytes. Although the sequencing depth of the current dataset provides a macro-level resolution, these annotations represent broad categorizations, providing descriptive transcriptional associations that serve as a foundational reference for future high-resolution functional validation.

### Preliminary assessment of druggable target genes and therapeutic compounds via DGIdb, DSigDB, and DrugBank

We identified 15 genes with reported or predicted druggability annotations from the DGIdb database, including *CFI*, *HAVCR2*, *KCNT2*, *KMT2E*, and *PTPRC* from the GWAS analysis and *LAMC2*, *PLTP*, *AGER*, *IGFBP6*, *VEGFA*, *TNFSF14*, *C3*, *BRD2*, *APOA5*, and *CSK* from the MR proteomics analysis. Among these, *LAMC2* and *PLTP*, previously prioritized as first-tier risk genes, were classified as druggable. Drug enrichment analysis using DSigDB revealed several therapeutic compounds significantly associated with the target gene sets (adjusted *P* value < 0.05; Additional file 2: Fig. S2). Cyclophosphamide, a broad-spectrum cytotoxic immunosuppressant with well-documented systemic toxicity, interacted with the highest number of prioritized genes (*CFI*, *PTPRC*, *LAMC2*, *BRD2*, and *VEGFA*) in the DSigDB enrichment analysis. Given the known toxicity profile of cyclophosphamide, this finding serves as a bioinformatic proof-of-concept, highlighting the immunological convergence of the gene targets and providing a conceptual framework for future, safer targeted interventions. A summary of current AMD therapies and their targets was generated after querying the DrugBank database (Additional file 1: Table S9). Anti-VEGF agents, including aflibercept, brolucizumab, faricimab, pegaptanib, and ranibizumab, all of which target VEGFA, are approved for the treatment of neovascular AMD. Additionally, the C3 inhibitor, pegcetacoplan, has been approved for treating geographic atrophy.

## Discussion

Our genetic investigation, including a meta-analysis of 28,543 AMD cases, identified 10 candidate loci of genome-wide significance associated with AMD. The identification of these loci, which were absent from the individual GWAS datasets, highlights the increased statistical power achieved through meta-analysis by pooling the sample size and reducing heterogeneity. The three previously reported loci (located near *PLEKHA1*/*ARMS2*/*HTRA1*, *CFI*, and *TIMP3*) further validated and replicated existing genetic associations, supporting the robustness of our meta-analytical approach and generalizability of these genetic signals. Our supplementary LDSC and locus-specific consistency analyses further reinforced this robustness by empirically demonstrating a highly shared genetic architecture across the distinct AMD stages included in our study [38]. Importantly, the remaining seven loci represent newly identified genetic associations discovered within the context of our meta-analysis. A comparison of effect sizes revealed that these novel variants exert phenotypic impacts (β ranging from 0.060 to 0.300) comparable to and, in instances such as for *DENND1B* (β = 0.300) and *PTPRC* (β = 0.202), exceeding that of the previously identified major risk locus, *ARMS2*/*HTRA1* (β = 0.243), replicated in our cohort. This robust effect size distribution confirms that our expanded multi-stage design successfully captured highly penetrant genetic drivers alongside essential moderate-effect modulators, substantially expanding the actionable genetic architecture of AMD.

Integrated MR and colocalization analyses identified 23 genetically supported plasma proteins; genetically predicted elevations in 11 proteins and reductions in 12 were associated with disease susceptibility. Although these findings establish robust genetically predicted associations between plasma proteins and AMD risk, further experimental validation is warranted to elucidate the precise mechanistic links. Multi-omics SMR analyses with HEIDI testing across protein, gene expression, and DNA methylation levels confirmed LAMC2 and PLTP as first-tier AMD risk factors. Mediation analysis offered exploratory insights into potential immune-related pathways linking circulating proteins to AMD; however, the small mediation proportions observed suggest that these immune traits are unlikely to represent the primary mechanisms underlying the protein–AMD associations. Collectively, our results provide a high-resolution, hypothesis-generating resource that paves the way for future mechanistic investigations into AMD pathogenesis. Our study expands the biological pathways for known AMD risk loci and highlights seven promising druggable targets for primary prevention, including five from GWAS and two from MR–colocalization analysis.

### Genetic insights into AMD pathogenesis: bridging complement pathways and immune-cell responses

Analysis of differentially expressed genes associated with risk variants revealed several pathways implicated in AMD pathogenesis. These findings offer insights into the genetic architecture of AMD and suggest that lymphocytes, particularly T and B cells, play pivotal roles in AMD immunopathology. Dysfunctional or aberrantly activated lymphocytes may exacerbate retinal inflammation, thereby driving AMD development. Significant enrichment of target genes within the complement system confirms that complement–immune interactions are critical for disease progression, particularly during early stages, such as drusen formation and blood–retina barrier breakdown, consistent with earlier reports by Hageman et al. [39].

Our MR-PheWAS revealed genetic correlations between candidate variants and established AMD risk factors. The rs10033900/CFI variant was linked to *CFH* and *HTRA1*, which are key AMD pathogenic genes. While prior studies have associated rs10033900 with AMD risk [40], our results indicate that its effect is predominantly genetic, suggesting high AMD specificity. Cardiometabolic factors, such as elevated blood pressure and cholesterol levels, have been linked to geographic atrophy [41], and rs11630478/EDC3 may mediate this relationship. Notably, rs3801281/KMT2E was associated with ultraviolet exposure and vitamin D levels, supporting the potential protective role of sun avoidance or supplementation [41]. The remaining variants, namely rs12141142/PTPRC, rs148136314/DENND1B, rs11134780/HAVCR2, and rs7289865/FBXO7, were implicated in immune regulation, coagulation, lung function, HbA1c, and stature, respectively, broadening our understanding of the multi-system regulatory network of AMD.

Screening of gene KO mouse models revealed that the ablation of *EDC3*, *KMT2E*, and *PLEKHA1* leads to visual impairments, supporting their roles in retinal function. Collectively, these pleiotropic associations offer a comprehensive trait profile that aligns with established AMD-related mechanisms, including complement activation, cardiometabolic profiles, immune regulation, and ultraviolet exposure. By underscoring the multifaceted pathways through which genetic variations may influence AMD susceptibility, these supplementary findings provide an essential systemic backdrop for evaluating the broader biological impact and potential safety considerations of the identified targets. Three candidate loci were of particular interest: rs12141142/PTPRC, rs3801281/KMT2E, and rs11134780/HAVCR2. Single-cell analysis demonstrated cell-type-specific expression changes for these genes in AMD retinas. Given that these descriptive observations were derived from a single scRNA-seq dataset, they provide a preliminary cell-type-specific context. Subsequent replication and functional validation in independent cohorts are essential to precisely define their mechanistic contributions to AMD pathogenesis. PTPRC (CD45), a key immune regulator, participates in early inflammatory responses, which is consistent with the reported secretion of G-CSF by choroidal CD45^+^ cells in patients with AMD [42]. KMT2E (MLL5) expression was significantly reduced in stem cells and monocytes; its loss decreases H3K4/K79 methylation, consequently disrupting CRX-CBP binding to photoreceptor gene promoters and impairing retinal function [43]. This observation aligns with early photoreceptor damage reported in patients with AMD [44], suggesting that reduced KMT2E expression may serve as an early AMD biomarker. HAVCR2 (TIM-3), an immune checkpoint molecule, induces Th1 apoptosis upon Gal-9 binding, thereby suppressing IFN-γ-mediated inflammation [45]. Given the Th1 cell enrichment and IFN-γ/IL-17 responses observed in patients with AMD [46], the TIM-3/Gal-9 axis may contribute to retinal immune homeostasis, potentially via Müller glial Gal-9 expression [47], thus warranting further mechanistic and therapeutic investigation.

The complement system, a key element of innate immunity, plays a central role in AMD pathogenesis through both local and systemic components. Single-cell sequencing indicated potential local functional roles of C3 within the retina, whereas multi-omics MR and colocalization analyses identified circulating C3d and CFHR3 as risk factors for AMD. C3d, a downstream product of C3, sustains immune activation by binding to complement receptors, thereby exacerbating chronic macular inflammation and serving as a systemic marker of long-term complement activation during AMD progression [48]. In contrast, CFHR3 is minimally expressed in the retina; however, elevated systemic levels competitively inhibit CFH, thereby promoting C3b deposition and dysregulating complement activation. High CFHR3 expression correlates with AMD progression, whereas its deletion may offer protective effects; CFHR3 may potentially interact with variants, such as CFH Y402H, to accelerate disease progression [49]. While current research emphasizes local complement activity, we propose that reducing circulating C3d or CFHR3 levels may offer a feasible strategy to slow AMD progression. Although the C3-targeting drug, pegcetacoplan, has shown efficacy in slowing geographic atrophy, its potent C3 inhibition may entail unforeseen immunological risks [50]. Our findings that C3d acts on myeloid and plasmacytoid dendritic cells and that CFHR3 expression correlates with increased NK-cell proportions suggest that these factors promote pathological immune-cell accumulation and inflammation. Thus, future therapies should aim not only to suppress complement activity but also to modulate immune-cell function for a more comprehensive control of AMD immunopathology.

### Candidate mechanisms in AMD: LAMC2 orchestrates an ECM–immune axis, whereas PLTP drives lipid–immune interplay

Laminin Subunit Gamma 2 (LAMC2), identified as a first-tier risk protein in our study, is recognized for its roles in immune regulation, angiogenesis, and extracellular matrix (ECM) remodeling, with well-established functions in cancer biology [51]. In AMD pathogenesis, the interaction between RPE cells and the ECM is essential for maintaining retinal structural integrity, and ECM disruption often leads to RPE dysfunction [52]. As a key ECM component, LAMC2 may promote AMD progression by altering retinal ECM structure and impairing RPE function. In addition to its ECM effects, the immunomodulatory role of LAMC2 is increasingly acknowledged [53]. Our analysis showed that LAMC2 levels correlated with proportional changes in Treg subsets, including CD28^+^ CD45RA^+^ CD8^dim^ T cells, activated and resting CD4^+^ Tregs, and secreting CD4^+^ Tregs. MR mediation analysis suggested that LAMC2 might be associated with changes in Treg-related immune traits, raising the hypothesis that LAMC2 influences AMD through immune modulation. Although the mediation proportions measured were modest, this exploratory insight highlights an adjunctive immunomodulatory axis that warrants further experimental validation alongside the primary ECM pathways in future studies.

LAMC2 may also contribute to neovascular AMD, a condition marked by pathological choroidal neovascularization and dysregulated immunity [54]. By modifying the ECM structure and influencing immune-cell behavior, LAMC2 could facilitate angiogenic factor expression and neovascularization, paralleling its pro-angiogenic role in tumor microenvironments. Our study is the first to delineate the significant role of LAMC2 in AMD through ECM remodeling and Treg-mediated immunomodulation, suggesting new directions for therapeutic development.

Our study also identified PLTP as a first-tier risk protein critical in AMD pathogenesis. PLTP mediates phospholipid transfer between lipoproteins and plays a key role in high-density lipoprotein assembly and remodeling. We and others have observed significantly elevated PLTP levels in the plasma of patients with AMD, suggesting that the protein contributes to AMD by disrupting retinal lipid homeostasis and promoting immune activation [55]. We further demonstrated that the underlying mechanism of PLTP involves not only lipid metabolic dysregulation but also inflammatory enhancement. Single-cell analysis showed that PLTP is upregulated in retinal monocytes, potentially driving pro-inflammatory activation, whereas its downregulation in astrocytes may impair their neuro-supportive functions. Collectively, these alterations promote AMD development. These findings suggest that PLTP may serve as a candidate plasma biomarker and thus warrant further investigation as a potential target for modulating lipid–immune interactions in AMD.

The therapeutic potential of targeting these pathways remains an important avenue for future clinical investigations. Notably, LAMC2 and PLTP did not show statistically significant cell-type-specific enrichment in our scRNA-seq analysis. This finding may reflect their roles as secreted proteins that act systemically or in a paracrine manner, functioning alongside localized transcriptional activities, and do not diminish the cumulative multi-omics evidence supporting their involvement in AMD pathogenesis.

### Cross-validation of AMD-associated targets and their therapeutic implications

Our study further substantiates the genetic basis of AMD through additional proteins and genes supported by prior evidence, including WARS1, BRD2, LBP, and polymorphic loci such as CSK (rs1378942), PILRA (rs10241492), and AGER (rs1800624; rs1800625). RBP1 deficiency has been linked to retinal dystrophy and early AMD pathology [56], whereas NID2 accumulation in the aging retina offers further mechanistic insight. These targets are implicated in the pathways involved in immunomodulation, complement activation, and lipid metabolism, suggesting opportunities for drug repurposing. For example, cyclophosphamide was computationally predicted to interact with multiple prioritized genes identified in the present study. Consequently, this specific druggability analysis functions as an initial hypothesis-generating step, outlining actionable molecular networks that could guide the subsequent discovery of highly specific and tolerable therapeutic agents [57]. Moreover, temporal gene expression dynamics may help identify key regulators at specific disease stages, supporting the development of staged intervention strategies.

## Strengths and limitations

This study has notable strengths, including the large sample size used, which enabled the robust identification of candidate AMD variants and genetically supported proteins via GWAS and MR. We implemented complementary gene prioritization strategies by integrating both local (nearest gene) and global (PoPS) methods. The pleiotropic effects of candidate variants were systematically characterized through MR-PheWAS, and the biological relevance of protein targets evaluated through an integrative pipeline spanning MR, colocalization, multi-omics SMR, gene enrichment, mouse KO model, and single-cell analyses. This multifaceted approach provided convergent evidence for the involvement of the identified proteins, with a particular focus on immunological mechanisms that add functional depth.

This study had several limitations that should be acknowledged. First, partial sample overlap exists both within our GWAS meta-analysis cohorts (e.g., the UK Biobank participants) and between the proteomic exposure (UKB-PPP) and outcome datasets. Although mitigated by distinct phenotypic definitions and rigorous sensitivity analyses, this overlap could theoretically bias causal estimates. However, several lines of evidence mitigate this concern, including the near-ideal LDSC intercept (0.9972), strong instrument strength (all F-statistics > 10), and highly consistent results validated in the independent FinnGen population. These additional sensitivity analyses support the robustness of our results against artificial inflation owing to sample overlap. Additionally, the predominantly European ancestry of the GWAS cohorts restricts the global generalizability of our findings. Second, although our MR framework predominantly utilized *cis*-pQTLs to tightly link genetic variants to protein expression, residual pleiotropy remains a potential confounding factor. Furthermore, the reliance on a limited number of instrumental SNPs for certain proteins may influence the statistical power of these specific genetically supported estimates. We deliberately adopted a colocalization threshold of PP.H4 > 0.5 to identify moderate evidence of shared genetically supported variants; therefore, targets meeting this permissive threshold warrant cautious interpretation and require rigorous downstream experimental validation. Third, defining candidate loci based solely on a 500-kb distance threshold may not fully account for long-range LD [25]; future conditional analyses adjusting for known index variants are needed to strictly confirm independence. Fourth, the mediation effects measured for the identified immune traits were modest, and these findings provide a conceptual framework that warrants further experimental exploration. Fifth, our assessment of mouse models primarily captured loss-of-function phenotypes. The evaluation of disease-relevant gain-of-function or dominant-negative mechanisms requires alternative knock-in models. Finally, standard GO enrichment may underrepresent highly relevant individual genes that do not co-aggregate into broader terms; however, we countered this by employing orthogonal, gene-centric multi-omics and single-cell profiling to ensure that critical targets, such as LAMC2 and PLTP, were robustly prioritized.

## Conclusion

In summary, our GWAS meta-analysis identified 10 candidate loci (including three previously reported and seven newly identified loci) and 23 plasma proteins with suggestive associations through MR proteomics. Mediation analysis offered exploratory insights into potential immune-related pathways linking circulating proteins to AMD. Collectively, these findings generated testable hypotheses for future mechanistic studies and highlighted candidate targets for further experimental evaluation. However, the proposed therapeutic implications remain speculative and require validation in preclinical and clinical settings.

## Supplementary Information

**Additional file 1.** **Table S1.** Summary of GWAS datasets included in the meta-analysis. **Table S2.** Sensitivity analyses for the two-step Mendelian randomization mediation analysis.** Table S3.** Previously reported AMD risk loci successfully replicated in the GWAS meta-analysis.** Table S4.** Conditional analysis (COJO) results for the newly identified AMD loci. **Table S5.** LDSC-based genetic correlation and heritability estimates for early and advanced AMD.** Table S6.** Consistency of effect estimates for lead variants across the meta-analysis and the independent FinnGen advanced AMD cohort.** Table S7.** MR-PheWAS results of newly discovered variants from the meta-analysis.** Table S8A.** The resuluts of proteome‑wide Mendelian randomization analysis between 1,811 cis-pQTLs of deCODE genetics and AMD.** Table S8B.** The results of proteome‑wide Mendelian randomization analysis between 1,113 cis-pQTLs of UKB-PPP and AMD.** Table S8C.** The F-statistics for genetic instruments for each protein of deCODE genetics.** Table S8D.** The F-statistics for genetic instruments for each protein of UKB-PPP.** Table S9.** Association analysis of gene colocalization with AMD for unique plasma proteins. **Table S10.** Reverse causal analysis: Steiger direction test results for plasma proteins in AMD.** Table S11.** Reverse causal analysis: Steiger filtering results for plasma proteins in AMD.** Table S12.** SMR analysis of 23 key proteins mapped to 22 genes across 49 tissues from the transcriptome and eQTLGen databases.** Table S13.** SMR and HEIDI analysis results for proteins associated with AMD.** Table S14.** Two-sample Mendelian randomization analysis of immune cell traits and AMD risk. **Table S15.** Phenotypic annotation of knockout mouse models of AMD-associated genes.** Table S16.** Differential expression of candidate genes in single-cell RNA sequencing data. **Table S17.** Five existing drug targets for AMD and their corresponding medications.

**Additional file 2.** **Figure S1.** Gene Ontology (GO) enrichment analysis of putative target genes identified by Mendelian randomization.** Figure S2.** Drug enrichment analysis for the prioritized druggable target genes.

## Data Availability

All data supporting the findings of this study are provided within the article and its supplementary tables. The original summary statistics from the AMD GWAS meta-analysis will be publicly available upon publication through the GWAS Catalog. All secondary data sources used in this study are publicly available through the following repositories: FinnGen (https://www.finngen.fi/en/access_results), deCODE Genetics (https://www.decode.com/summarydata/), UK Biobank Pharma Proteomics Project (https://www.synapse.org/Synapse:syn51364943), GTEx Portal (https://gtexportal.org/home/datasets), eQTLGen Consortium (https://eqtlgen.org/cis-eqtls.html), GWAS Catalog immune traits (https://www.ebi.ac.uk/gwas/), and GEO accession GSE203499 for single-cell RNA sequencing data. Analysis code for this study is available at GitHub repository [URL will be provided upon acceptance]. The Mendelian randomization analyses were conducted using the TwoSampleMR R package (https://github.com/MRCIEU/TwoSampleMR), and colocalization analyses were performed using the coloc R package (https://github.com/chr1swallace/coloc). All software used are publicly available. Further information and requests for resources should be directed to the corresponding author.

## Abbreviations

AMD: Age-related macular degeneration
BP: Biological process
CC: Cellular component
*cis*-eQTL: *cis*-acting expression quantitative trait loci
*cis*-pQTL: *cis*-acting protein quantitative trait loci
COJO: Conditional and joint analysis
DGIdb: Drug–Gene Interaction Database
DSigDB: Drug Signatures Database
ECM: Extracellular matrix
eQTL: Expression quantitative trait loci
FDR: False discovery rate
GO: Gene ontology
GWAS: Genome-wide association study
HEIDI: Heterogeneity in dependent instruments test
IAMDGC: International Age-Related Macular Degeneration Genomics Consortium
IV: Instrumental variable
IVW: Inverse-variance weighted
KO: Knockout
LD: Linkage disequilibrium
LDSC: Linkage disequilibrium score regression
MAF: Minor allele frequency
MF: Molecular function
MGI: Mouse Genome Informatics
mQTL: Methylation quantitative trait locus
MR: Mendelian randomization
MR-PheWAS: Mendelian randomization phenome-wide association study
OR: Odds ratio
PoPS: Polygenic priority score
PP.H4: Posterior probability for hypothesis 4
pQTL: Protein quantitative trait loci
RPE: Retinal pigment epithelium
scRNA-seq: Single-cell RNA sequencing
SE: Standard error
SMR: Summary-data-based Mendelian randomization
SNP: Single nucleotide polymorphism
Treg: Regulatory T cell
UKB-PPP: UK Biobank Pharma Proteomics Project

## References

[1] Thomas CJ, Mirza RG, Gill MK. Age-related macular degeneration. Med Clin North Am. 2021;105(3):473–91. 33926642 10.1016/j.mcna.2021.01.003

[2] Fabre M, Mateo L, Lamaa D, Baillif S, Pagès G, Demange L, et al. Recent advances in age-related macular degeneration therapies. Molecules. 2022;27(16):5089. 36014339 10.3390/molecules27165089 PMC9414333

[3] Liu S, Chen C, Ning J, Ding P, Chen Y, Xie T, et al. Association between early inflammation and neurodegeneration in diabetic retinopathy: a narrative review. Vis Neurosci. 2025;42:e012. 10.48130/vns-0025-0012.

[4] Fritsche LG, Fariss RN, Stambolian D, Abecasis GR, Curcio CA, Swaroop A. Age-related macular degeneration: genetics and biology coming together. Annu Rev Genom Hum Genet. 2014;15:151–71. 10.1146/annurev-genom-090413-025610 PMC4217162 24773320

[5] Smith GD, Ebrahim S. Mendelian randomization: prospects, potentials, and limitations. Int J Epidemiol. 2004;33(1):30–42. 15075143 10.1093/ije/dyh132

[6] Fritsche LG, Igl W, Bailey JNC, Grassmann F, Sengupta S, Bragg-Gresham JL, et al. A large genome-wide association study of age-related macular degeneration highlights contributions of rare and common variants. Nat Genet. 2016;48(2):134–43. 26691988 10.1038/ng.3448 PMC4745342

[7] He W, Han X, Ong JS, Wu Y, Hewitt AW, Mackey DA, et al. Genome-wide meta-analysis identifies risk loci and improves disease prediction of age-related macular degeneration. Ophthalmology. 2024;131(1):16–29. 37634759 10.1016/j.ophtha.2023.08.023

[8] Han X, Lains I, Li J, Li J, Chen Y, Yu B, et al. Integrating genetics and metabolomics from multi-ethnic and multi-fluid data reveals putative mechanisms for age-related macular degeneration. Cell Rep Med. 2023;4(7):101085. 37348500 10.1016/j.xcrm.2023.101085 PMC10394104

[9] Stradiotto E, Allegrini D, Fossati G, Raimondi R, Sorrentino T, Tripepi D, et al. Genetic aspects of age-related macular degeneration and their therapeutic potential. Int J Mol Sci. 2022;23(21):13280. 36362067 10.3390/ijms232113280 PMC9653831

[10] Zheng J, Haberland V, Baird D, Walker V, Haycock PC, Hurle MR, et al. Phenome-wide Mendelian randomization mapping the influence of the plasma proteome on complex diseases. Nat Genet. 2020;52(10):1122–31. 32895551 10.1038/s41588-020-0682-6 PMC7610464

[11] Reay WR, Cairns MJ. Advancing the use of genome-wide association studies for drug repurposing. Nat Rev Genet. 2021;22(10):658–71. 34302145 10.1038/s41576-021-00387-z

[12] Wu J, Sun X. Complement system and age-related macular degeneration: drugs and challenges. Drug Des Devel Ther. 2019;13:2413–25. 31409975 10.2147/DDDT.S206355 PMC6650090

[13] Jiang L, Zheng Z, Fang H, Yang J. A generalized linear mixed model association tool for biobank-scale data. Nat Genet. 2021;53(11):1616–21. 34737426 10.1038/s41588-021-00954-4

[14] Winkler TW, Grassmann F, Brandl C, Kiel C, Günther F, Strunz T, et al. Genome-wide association meta-analysis for early age-related macular degeneration highlights novel loci and insights for advanced disease. BMC Med Genomics. 2020;13:120. 32843070 10.1186/s12920-020-00760-7 PMC7449002

[15] Kurki MI, Karjalainen J, Palta P, Sipilä TP, Kristiansson K, Donner KM, et al. FinnGen provides genetic insights from a well-phenotyped isolated population. Nature. 2023;613(7944):508–18. 36653562 10.1038/s41586-022-05473-8 PMC9849126

[16] Lin DY, Sullivan PF. Meta-analysis of genome-wide association studies with overlapping subjects. Am J Hum Genet. 2009;85(6):862–72. 20004761 10.1016/j.ajhg.2009.11.001 PMC2790578

[17] Willer CJ, Li Y, Abecasis GR. METAL: fast and efficient meta-analysis of genomewide association scans. Bioinformatics. 2010;26(17):2190–1. 20616382 10.1093/bioinformatics/btq340 PMC2922887

[18] Bulik-Sullivan BK, Loh PR, Finucane HK, Ripke S, Yang J, Schizophrenia Working Group of the Psychiatric Genomics Consortium, et al. LD score regression distinguishes confounding from polygenicity in genome-wide association studies. Nat Genet. 2015;47(3):291–5. 25642630 10.1038/ng.3211 PMC4495769

[19] Ferkingstad E, Sulem P, Atlason BA, Sveinbjornsson G, Magnusson MI, Styrmisdottir EL, et al. Large-scale integration of the plasma proteome with genetics and disease. Nat Genet. 2021;53(12):1712–21. 34857953 10.1038/s41588-021-00978-w

[20] Võsa U, Claringbould A, Westra HJ, Bonder MJ, Deelen P, Zeng B, et al. Large-scale cis- and trans-eQTL analyses identify thousands of genetic loci and polygenic scores that regulate blood gene expression. Nat Genet. 2021;53(9):1300–10. 34475573 10.1038/s41588-021-00913-z PMC8432599

[21] GTEx Consortium. The GTEx Consortium atlas of genetic regulatory effects across human tissues. Science. 2020;369(6509):1318–30. 32913098 10.1126/science.aaz1776 PMC7737656

[22] McRae AF, Marioni RE, Shah S, Yang J, Powell JE, Harris SE, et al. Identification of 55,000 replicated DNA methylation QTL. Sci Rep. 2018;8(1):17605. 30514905 10.1038/s41598-018-35871-w PMC6279736

[23] Orrù V, Steri M, Sidore C, Marongiu M, Serra V, Olla S, et al. Complex genetic signatures in immune cells underlie autoimmunity and inform therapy. Nat Genet. 2020;52(10):1036–45. 32929287 10.1038/s41588-020-0684-4 PMC8517961

[24] Weeks EM, Ulirsch JC, Cheng NY, Trippe BL, Fine RS, Miao J, et al. Leveraging polygenic enrichments of gene features to predict genes underlying complex traits and diseases. Nat Genet. 2023;55(8):1267–76. 37443254 10.1038/s41588-023-01443-6 PMC10836580

[25] Rasooly D, Peloso GM, Pereira AC, Dashti H, Giambartolomei C, Wheeler E, et al. Genome-wide association analysis and mendelian randomization proteomics identify drug targets for heart failure. Nat Commun. 2023;14(1):3826. 37429843 10.1038/s41467-023-39253-3 PMC10333277

[26] Ying H, Wu X, Jia X, Yang Q, Liu H, Zhao H, et al. Single-cell transcriptome-wide mendelian randomization and colocalization reveals immune-mediated regulatory mechanisms and drug targets for COVID-19. EBioMedicine. 2025;113:105596. 39933264 10.1016/j.ebiom.2025.105596 PMC11867302

[27] Burgess S, Davey Smith G, Davies NM, Dudbridge F, Gill D, Glymour MM, et al. Guidelines for performing mendelian randomization investigations: update for summer 2023. Wellcome Open Res. 2019;4:186. 32760811 10.12688/wellcomeopenres.15555.1 PMC7384151

[28] Yuan S, Xu F, Li X, Chen J, Zheng J, Mantzoros CS, et al. Plasma proteins and onset of type 2 diabetes and diabetic complications: proteome-wide mendelian randomization and colocalization analyses. Cell Rep Med. 2023;4(9):101174. 37652020 10.1016/j.xcrm.2023.101174 PMC10518626

[29] Chen J, Ruan X, Sun Y, Lu S, Hu S, Yuan S, et al. Multi-omic insight into the molecular networks of mitochondrial dysfunction in the pathogenesis of inflammatory bowel disease. EBioMedicine. 2024;99:104934. 38103512 10.1016/j.ebiom.2023.104934 PMC10765009

[30] Zhu Z, Zhang F, Hu H, Bakshi A, Robinson MR, Powell JE, et al. Integration of summary data from GWAS and eQTL studies predicts complex trait gene targets. Nat Genet. 2016;48(5):481–7. 27019110 10.1038/ng.3538

[31] Chen L, Zhang YH, Wang S, Zhang Y, Huang T, Cai YD. Prediction and analysis of essential genes using the enrichments of gene ontology and KEGG pathways. PLoS One. 2017;12(9):e0184129. 28873455 10.1371/journal.pone.0184129 PMC5584762

[32] Zhou Y, Zhou B, Pache L, Chang M, Khodabakhshi AH, Tanaseichuk O, et al. Metascape provides a biologist-oriented resource for the analysis of systems-level datasets. Nat Commun. 2019;10(1):1523. 30944313 10.1038/s41467-019-09234-6 PMC6447622

[33] Hao Y, Stuart T, Kowalski MH, Choudhary S, Hoffman P, Hartman A, et al. Dictionary learning for integrative, multimodal and scalable single-cell analysis. Nat Biotechnol. 2024;42(2):293–304. 37231261 10.1038/s41587-023-01767-y PMC10928517

[34] Aran D, Looney AP, Liu L, Wu E, Fong V, Hsu A, et al. Reference-based analysis of lung single-cell sequencing reveals a transitional profibrotic macrophage. Nat Immunol. 2019;20(2):163–72. 30643263 10.1038/s41590-018-0276-y PMC6340744

[35] Kanda A, Chen W, Othman M, Branham KEH, Brooks M, Khanna R, et al. A variant of mitochondrial protein LOC387715/ARMS2, not HTRA1, is strongly associated with age-related macular degeneration. Proc Natl Acad Sci. 2007;104(41):16227–32. 17884985 10.1073/pnas.0703933104 PMC1987388

[36] Yang Z, Camp NJ, Sun H, Tong Z, Gibbs D, Cameron DJ, et al. A variant of the HTRA1 gene increases susceptibility to age-related macular degeneration. Science. 2006;314(5801):992–3. 17053109 10.1126/science.1133811

[37] Jakobsdottir J, Conley YP, Weeks DE, Mah TS, Ferrell RE, Gorin MB. Susceptibility genes for age-related maculopathy on chromosome 10q26. Am J Hum Genet. 2005;77(3):389–407. 16080115 10.1086/444437 PMC1226205

[38] Winkler TW, Day FR, Croteau-Chonka DC, Wood AR, Locke AE, Mägi R, et al. Quality control and conduct of genome-wide association meta-analyses. Nat Protoc. 2014;9(5):1192–212. 24762786 10.1038/nprot.2014.071 PMC4083217

[39] den Hollander AI, Mullins RF, Orozco LD, Voigt AP, Chen HH, Strunz T, et al. Systems genomics in age-related macular degeneration. Exp Eye Res. 2022;225:109248. 36108770 10.1016/j.exer.2022.109248 PMC10150562

[40] Yu QQ, Yao Y, Zhu Jing, Bao X, Xie TH, Sun C, et al. Nonsynonymous single nucleotide polymorphisms in the complement component 3 gene are associated with risk of age-related macular degeneration: a meta-analysis. Gene. 2015;561(2):249–55. 25688879 10.1016/j.gene.2015.02.039

[41] Zhong P, Tan S, Zhu Z, Bulloch G, Long E, Huang W, et al. Metabolomic phenotyping of obesity for profiling cardiovascular and ocular diseases. J Transl Med. 2023;21(1):384. 37308902 10.1186/s12967-023-04244-x PMC10262415

[42] Mulfaul K, Khan AH, Schwarte SG, Voigt AP, Moore RF, Potempa LA, et al. Elevation of granulocyte colony stimulating factor in human AMD donor RPE-choroid. Invest Ophthalmol Vis Sci. 2024;65(14):15. 39641748 10.1167/iovs.65.14.15 PMC11629913

[43] Zhang X, Zhang BW, Xiang L, Wu H, Alexander SAS, Zhou P, et al. MLL5 is involved in retinal photoreceptor maturation through facilitating CRX-mediated photoreceptor gene transactivation. iScience. 2022;25(4):104058. 35359806 10.1016/j.isci.2022.104058 PMC8961232

[44] Guymer RH, Silva R, Ghadessi M, Leal S, Gashaw I, Damask A, et al. ANO2 genetic variants and anti-VEGF treatment response in neovascular AMD: a pharmacogenetic substudy of VIEW 1 and VIEW 2. Invest Ophthalmol Vis Sci. 2024;65(8):17. 38980270 10.1167/iovs.65.8.17 PMC11244643

[45] Zhu C, Anderson AC, Schubart A, Xiong H, Imitola J, Khoury SJ, et al. The Tim-3 ligand galectin-9 negatively regulates T helper type 1 immunity. Nat Immunol. 2005;6(12):1245–52. 16286920 10.1038/ni1271

[46] Chen J, Wang W, Li Q. Increased Th1/Th17 responses contribute to low-grade inflammation in age-related macular degeneration. Cell Physiol Biochem. 2017;44(1):357–67. 29132135 10.1159/000484907

[47] Quinn J, Salman A, Paluch C, Jackson-Wood M, McClements ME, Luo J, et al. Single-cell transcriptomic analysis of retinal immune regulation and blood-retinal barrier function during experimental autoimmune uveitis. Sci Rep. 2024;14(1):20033. 39198470 10.1038/s41598-024-68401-y PMC11358488

[48] Armento A, Ueffing M, Clark SJ. The complement system in age-related macular degeneration. Cell Mol Life Sci. 2021;78(10):4487–505. 33751148 10.1007/s00018-021-03796-9 PMC8195907

[49] Enzbrenner A, Zulliger R, Biber J, Pousa AMQ, Schäfer N, Stucki C, et al. Sodium iodate-induced degeneration results in local complement changes and inflammatory processes in murine retina. Int J Mol Sci. 2021;22(17):9218. 34502128 10.3390/ijms22179218 PMC8431125

[50] Śpiewak D, Drzyzga Ł, Dorecka M, Wyględowska-Promieńska D. Summary of the therapeutic options for patients with dry and neovascular AMD. J Clin Med. 2024;13(14):4227. 39064267 10.3390/jcm13144227 PMC11278184

[51] Erice O, Narayanan S, Feliu I, Entrialgo-Cadierno R, Malinova A, Vicentini C, et al. LAMC2 regulates key transcriptional and targetable effectors to support pancreatic cancer growth. Clin Cancer Res. 2023;29(6):1137–54. 36607777 10.1158/1078-0432.CCR-22-0794

[52] Cheng L, Liu X, Lv K, Zhang Y, Jin P, Qian T, et al. Neuropeptide VF promotes hypoxia-inducible factor-1α expression in age-related macular degeneration via the aldehyde dehydrogenase 2/D-glycerate axis. Vis Neurosci. 2025;42:e024.

[53] Hussain AA, Lee Y. Extracellular matrix (ECM) aging in the retina: the role of matrix metalloproteinases (MMPs) in Bruch’s membrane pathology and age-related macular degeneration (AMD). Biomolecules. 2025;15(8):1059. 40867504 10.3390/biom15081059 PMC12383675

[54] Heloterä H, Kaarniranta K. A linkage between angiogenesis and inflammation in neovascular age-related macular degeneration. Cells. 2022;11(21):3453. 36359849 10.3390/cells11213453 PMC9654543

[55] Kim HJ, Ahn SJ, Woo SJ, Hong HK, Suh EJ, Ahn J, et al. Proteomics-based identification and validation of novel plasma biomarkers phospholipid transfer protein and mannan-binding lectin serine protease-1 in age-related macular degeneration. Sci Rep. 2016;6:32548. 27605007 10.1038/srep32548 PMC5015054

[56] Abcouwer SF, Miglioranza Scavuzzi B, Kish PE, Kong D, Shanmugam S, Le XA, et al. The mouse retinal pigment epithelium mounts an innate immune defense response following retinal detachment. J Neuroinflammation. 2024;21(1):74. 38528525 10.1186/s12974-024-03062-2 PMC10964713

[57] Luong SN, Isaacs A, Liu Z, Sin FE, Giles I. A systematic review and meta-analysis of the gonadotoxic effects of cyclophosphamide and benefits of gonadotropin releasing hormone agonists (GnRHa) in women of child-bearing age with autoimmune rheumatic disease. Expert Rev Clin Immunol. 2020;16(3):321–33. 32005081 10.1080/1744666X.2020.1724091

