# Supplementary material for: Integrative genomic and proteomic analyses identify candidate genetic loci and genetically supported proteins in age-related macular degeneration
Source: Eye Vis (Lond). 2026 Aug 14;13:35. doi: 10.1186/s40662-026-00502-3 (PMC13476935; doi:10.1186/s40662-026-00502-3)
Supplement: Supplementary file 1 [file 40662_2026_502_MOESM2_ESM.docx]

**Supplementary figure**

**Supplementary Figure 1: Gene Ontology (GO) enrichment analysis of putative target genes identified by Mendelian randomization.** The bar graph displays significantly enriched GO terms across the three ontologies: Biological Process (BP), Cellular Component (CC), and molecular function (MF). The enrichment score (x-axis) represents the significance level. In the BP domain, target genes were significantly enriched for terms including positive regulation of leukocyte migration and positive regulation of chemotaxis. The most significantly enriched term in the CC domain was high-density lipoprotein particle. Within the MF domain, the most prominent enrichment was for fibronectin binding.

# Supplementary Figure 2. Drug enrichment analysis for the prioritized druggable target genes.

**a** Bar plot showing the top significant compounds enriched from the DsigDB database that interact with the identified target genes. The x-axis (Count) represents the number of target genes interacting with each compound. Cyclophosphamide, which interacts with the highest number of genes (including CFI, PTPRC, LAMC2, BRD2, and VEGFA), is highlighted. **b** Network or bubble plot visualizing the significant associations between the candidate compounds (y-axis) and the target gene sets. The significance of the enrichment is represented by the adjusted *P* value (*P* adjust), with a cutoff of < 0.05.
